# SWISH-X, an Expanded Approach to Detect Cryptic Pockets in Proteins and at Protein–Protein Interfaces

**DOI:** 10.1021/acs.jctc.3c01318

**Published:** 2024-04-02

**Authors:** Alberto Borsatto, Eleonora Gianquinto, Valerio Rizzi, Francesco Luigi Gervasio

**Affiliations:** † School of Pharmaceutical Sciences, University of Geneva, 1205 Geneva, Switzerland; ‡ Institute of Pharmaceutical Sciences of Western Switzerland, University of Geneva, 1205 Geneva, Switzerland; ¶ Swiss Institute of Bioinformatics, 1015 Lausanne, Switzerland; § Department of Drug Science and Technology, University of Turin, 10125 Turin, Italy; ∥ Department of Chemistry, University College London, WC1 H0AJ London, United Kingdom; ⊥ Institute of Structural and Molecular Biology, University College London, WC1E7JE London, United Kingdom

## Abstract

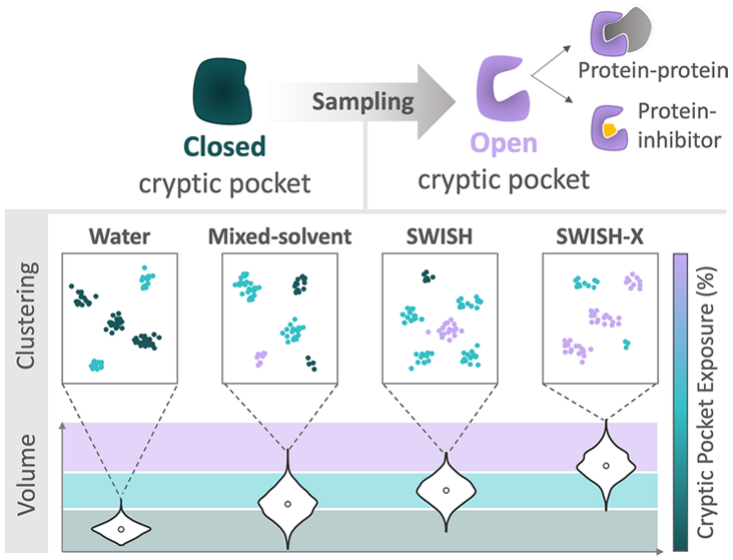

Protein–protein interactions mediate most molecular processes in the cell, offering a significant opportunity to expand the set of known druggable targets. Unfortunately, targeting these interactions can be challenging due to their typically flat and featureless interaction surfaces, which often change as the complex forms. Such surface changes may reveal hidden (cryptic) druggable pockets. Here, we analyze a set of well-characterized protein–protein interactions harboring cryptic pockets and investigate the predictive power of current computational methods. Based on our observations, we developed a new computational strategy, SWISH-X (SWISH Expanded), which combines the established cryptic pocket identification capabilities of SWISH with the rapid temperature range exploration of OPES MultiThermal. SWISH-X is able to reliably identify cryptic pockets at protein–protein interfaces while retaining its predictive power for revealing cryptic pockets in isolated proteins, such as TEM-1 β-lactamase.

## 1 Introduction

Protein–protein interactions (PPIs) mediate a variety of biological processes from signal transduction to enzymatic regulation, forming an intricate network of interactions known as the “interactome”. The human interactome is estimated in the hundreds of thousands of different protein–protein interactions, each mediating a specific biological process.^1^ These interactions, when disrupted, can lead to a variety of human diseases, making them highly promising targets for drug development.^2^ Over the past few decades, considerable effort has been devoted to investigating the druggability of PPIs, which has proven to be a challenging research endeavor.^3^

There are several examples of PPI modulators, including peptides, antibodies, and small molecules, each with certain advantages and limitations.^4^ Peptide modulators promise greater specificity, can be designed to mimic one of the partner proteins, and have reduced toxicity. However, the interacting amino acids of a PPI are often not contiguous in the protein sequence, making these interactions difficult to replicate with synthetic peptides. In addition, peptides are limited by their short half-life, potential immunogenicity, and low membrane permeability.^5^ Antibodies, due to their unique characteristics, are particularly promising as modulators of protein–protein interactions. However, they are expensive to produce and mostly limited to targeting proteins that are secreted or located on the cell membrane.^6^

Small molecules offer significant opportunities for PPIs’ targeting. In recent years, a number of small molecules have been approved or entered clinical trials,^4^ illustrating the feasibility of targeting PPIs with this approach. However, significant challenges remain in the development of small molecule drugs that target PPIs, mainly due to the difficulty of finding suitable binding sites on the typically flat and featureless protein–protein interaction surfaces. Despite the typical large interaction surface (1500–3000 Å^2^), not all interface residues equally contribute to the free energy of binding. It has been shown that the interaction of a subset of hot-spot amino acids accounts for most of the binding free energy.^7,8^ Most of the hot-spots in a PPI are typically located at or near the interface, and various computational methods have been developed to identify druggable hot-spots.^9^ Therefore, small drug-like molecules that selectively bind to these hot-spots might effectively modulate a target PPI interface.^10,11^ However, finding a suitable pocket to accommodate such molecules can be challenging as conventional, stable cavities are often lacking in the structures of the individual partner proteins. Alternatively, cryptic pockets that form at the PPI interface may provide novel small-molecule-mediated targeting opportunities.

Cryptic binding sites are dynamic protein cavities that are undetectable in the ligand-free state and become apparent only upon ligand binding. The mechanisms governing the formation of cryptic pockets are often unclear and depend on the system under consideration. The free energy cost associated with the opening of these hidden sites varies according to the structural rearrangement required to expose the pocket. Typical structural changes associated with cryptic pocket formation are lateral chain rotations, loop motions, secondary structure changes, and interdomain motions.^12^ Due to their highly dynamic and elusive nature, the identification of cryptic pockets in single proteins and at PPI interfaces has proved challenging, often resulting from serendipitous discovery in experimentally determined protein structures.^13^ Despite the challenges in detecting cryptic pockets, several examples of small molecules targeting cryptic binding sites have been reported, showing that cryptic pockets are valuable candidates for expanding the set of known druggable targets.^14,15^

In recent years, a number of computational approaches have been developed with the aim of detecting cryptic binding sites, including machine learning-based methods,^16,17^ mixed-solvent molecular dynamics (MD) simulations,^18−21^ Markov state models,^22,23^ and Hamiltonian replica exchange techniques like SWISH (Sampling Water Interfaces through Scaled Hamiltonians).^24,25^ The latter, developed by our group, has proven to be effective in sampling the opening of cryptic pockets in various targets when combined with organic probes. Notable examples of combining both experiments and simulations to validate cryptic pockets have also been proposed, showing the promise of multifaceted approaches.^26,27^ However, even with SWISH and mixed-solvents, sampling of some cryptic sites associated with complex structural rearrangements remains challenging.

Here, we show that neither the original SWISH nor mixed-solvent MD is always effective in sampling the known cavities forming at a number of PPI interfaces. Thus, to overcome the limitations of current strategies, we combine SWISH with a recently developed On-the-fly Probability Enhanced Sampling (OPES) variant, OPES MultiThermal, to further enhance the sampling of the slow degrees of freedom linked to the formation of such cavities.^28−30^ We test the new method, which we named SWISH-X (SWISH Expanded), on a relevant and diverse set of PPIs known to harbor cryptic pockets for which liganded structures are available. We find that the performance of SWISH-X is significantly better than those of both the original SWISH and mixed-solvent simulations for complex systems. We also test the approach on TEM-1 beta-lactamase (β-lactamase), an established model system for studying cryptic pockets, and show how it retains the ability to sample the opening of the nontrivial cryptic cavity of the enzyme. Finally, we propose a clustering-based protocol for analyzing the resulting trajectories, which can be applied to study the conformational ensemble of any pocket structure.

## 2 Methods

In this study, we examined specific protein–protein interactions that are regulated by inhibitors. These inhibitors bind to one participant in the PPI (the target protein), effectively mimicking the role of the other participant. When selecting target proteins involved in PPIs, we considered targets for which structural information was available for the protein in both its unbound and its inhibitor-bound states. Next, we evaluated the crypticity of the binding site by comparing the structures of the unbound (apo) and bound (holo) states of the targets. Overall, four target proteins harboring cryptic pockets at the protein–protein interface were selected, namely Bcl-X_*L*_, IL-2, MDM2, and HPV-11 E2 (Table 1 and Supplementary Section 1.1). The opening of these cryptic sites requires different types of conformational changes, including an increasing number of lateral chain rotations in the case of IL-2, MDM2, and HPV-11 E2 and substantial secondary structure changes in the case of Bcl-X_*L*_. We also included TEM-1 β-lactamase in our test set as an additional example of cryptic pocket opening associated with a well-characterized secondary structure rearrangement. This enzyme is not involved in any known protein–protein interactions; therefore, its inclusion increases the diversity of our test set to both individual proteins and PPIs.

**Table 1:** Information about the Simulated Target Proteins^a^

**Target Protein**	**PPI**	**Biological Assembly**	**PDB ID apo state**	**PDB ID holo-like state**	**Ligand in holo-like structure**
TEM-1 β-lactamase	-	monomer	1JWP (1.75 Å)	1PZO (1.90 Å)	Small-molecule allosteric inhibitor
Bcl-X_*L*_	Bcl-X_*L*_/Bak	homodimer	1R2D (1.95 Å)	4C52 (2.05 Å)	Small-molecule PPI inhibitor
IL-2	IL-2/IL-2Rα	monomer	1M47 (1.99 Å)	1PY2 (2.80 Å)	Small-molecule PPI inhibitor
MDM2	MDM2/p53	monomer	1Z1M (b)	5LAV (1.73 Å)	Small-molecule PPI inhibitor
HPV-11 E2	HPV-11 E2/E1	monomer	1R6K (2.50 Å)	1R6N (2.40 Å)	Small-molecule PPI inhibitor

a X-ray structures are reported with their resolution in parentheses.

b = NMR structure.

**Figure 1:**
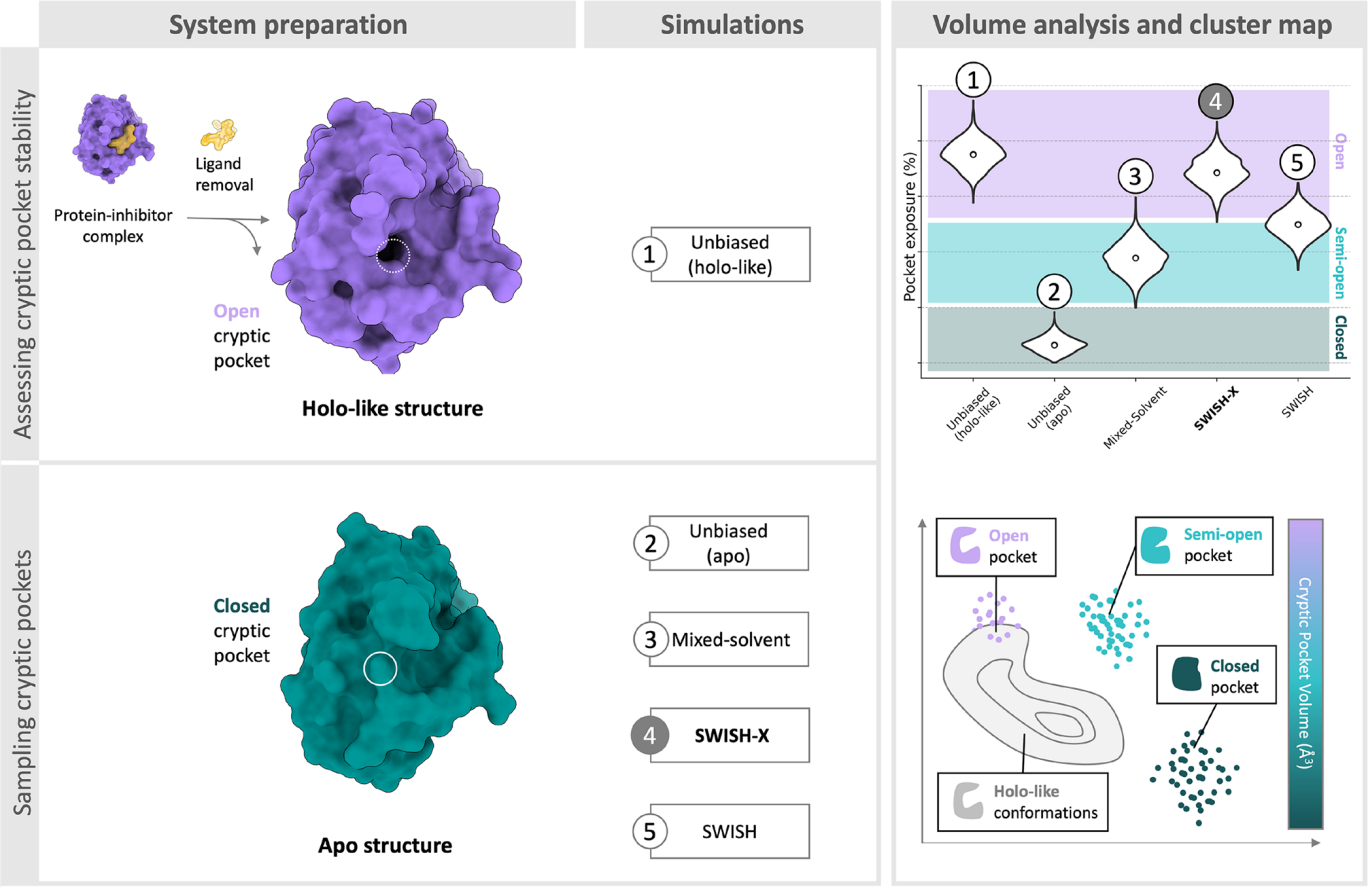
Simulation strategies and cluster map analysis for cryptic sites’ identification. First, we obtained the dynamics of the target protein in its holo-like state. Next, we employed various simulation protocols to reveal the cryptic site in the apo state of the same target protein. We then projected the obtained holo-like and apo structures, generated via a specific simulation protocol, into a contact-based t-SNE space. Clusters within this t-SNE space correspond to different configurations of the cryptic pockets under investigation.

We investigated the dynamics of the cryptic pocket harbored by each selected target protein using different simulation methods (Figure 1). First, we evaluated the stability of the open configuration of the cryptic pocket by running three independent unbiased MD simulations in water, each for 500 ns. The evaluation was performed after removing the ligand from the holo structure of the target protein, which is hereafter referred to as the holo-like or open-like state. We then performed three 500 ns long unbiased MD simulations starting from the apo structure of the protein - a closed pocket state - in water to test whether unbiased MD is sufficient to capture the conformational change associated with the opening of the cryptic site. Finally, we ran mixed-solvent, SWISH and SWISH-X simulations to assess their capability to retrieve the open conformation of the cryptic pocket from its closed configuration and to compare their performance. The details of the different simulation protocols are outlined in sections 2.1 and 2.2.

Additionally, we generated cluster maps to assess the diverse conformations of the pockets sampled during the various simulations starting from the apo structures. The cluster maps were obtained by using a clustering protocol that comprises three steps: Principal Component Analysis (PCA),^31,32^ t-Distributed Stochastic Neighbor Embedding (t-SNE),^33^ and Hierarchical Density-Based Spatial Clustering of Applications with Noise (HDBSCAN).^34^ By clustering the t-SNE space projections with HDBSCAN, we obtained separate clusters corresponding to different configurations of the cryptic pocket. We highlighted the region of t-SNE space sampled by the holo-like simulations with contour kernel density lines. Cluster points close to or within the contour of the holo-like configurations generally resembled open configurations of the cryptic pocket. Distant clusters, on the other hand, corresponded to different conformations of the pocket, predominantly closed configurations. Analyzing the resulting cluster maps, in combination with information on pocket volume, provided insights into how effectively the different simulations captured physically meaningful conformations of the open state of the cryptic pocket in each presented protein system. Further details on system preparation, equilibration strategies, and pocket analyses presented in this work can be found in Supplementary Section 1.

### 2.1 SWISH Expanded

In previous work, unbiased simulations captured the opening of known cryptic sites in several protein targets.^35^ When the free energy associated with cryptic pocket formation is not too high, the pocket’s opening can be successfully sampled by unbiased simulations of the unliganded target. However, identifying cryptic binding pockets can be frustrated by the short time scales accessible through unbiased MD simulations. The time-scale problem hinders the sampling of conformational rearrangements with significant activation energy barriers such as secondary structure changes and interdomain motions.

Enhanced sampling methods, like SWISH, have proven effective at overcoming the time-scale limitations of unbiased simulations, and they have indeed been successful in exposing nontrivial cryptic pockets.

SWISH is a Hamiltonian replica-exchange method that accelerates the sampling of cryptic pockets.^24,25^ In a SWISH simulation, the nonbonded interactions between water molecules and apolar atoms of the protein are modified by a scaling factor λ. The corresponding nonbonded component of the potential energy (*U*_nonb_) reads
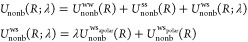
1where the ww term indicates water–water interactions, the ws term indicates water–solute interactions, the ss term indicates solute–solute interactions, and *R* represents the system’s coordinates.

For increasing values of λ, water’s properties are shifted toward higher affinity values for apolar protein atoms. In doing so, SWISH induces the opening of hydrophobic cavities that can be later stabilized by organic fragments, if included in the simulation. Typically, the first replica is not scaled, and increasing lambda factors are selected for higher replicas. Striking a balance in the choice of the scaling factor range is crucial, as the highest replica should display significant fluctuations while preserving the structural integrity of the protein.

SWISH simulations provide the best results when they are combined with a mixed-solvent approach. Organic cosolvent molecules, also due to their larger molecular size, act to stabilize the pockets induced by the scaled water–protein interactions at higher λ factors. Specifically, at higher λ scaling, the water molecules display a higher affinity for the apolar protein atoms. This increased affinity facilitates the opening of the cryptic sites. When these structures are exchanged back to replicas characterized by intermediate λ factors, the organic probes can now exploit the opening to bind and further stabilize the cavity.^24^ However, even with SWISH simulations in the presence of cosolvent molecules, the opening of cryptic pockets associated with higher energy barriers remains challenging. This is primarily because, when the conformational change is substantial and the associated free energy cost is high, a slight increase in the interactions between water molecules and the site is insufficient to sample the formation of the cryptic cavity within the time scale accessible by MD simulations.

To help overcome the free energy barriers associated with cryptic pocket formation, we have combined SWISH with OPES MultiThermal and named this approach SWISH Expanded (SWISH-X). OPES MultiThermal^29^ is in a way similar to simulated tempering techniques,^36−39^ allowing the system to effectively sample a selected temperature range. By enhancing the system’s potential energy (*U*) fluctuations, OPES MultiThermal enables the exploration of a multicanonical ensemble spanning temperatures denoted as *T*_*j*_, where *j* spans the defined temperature range [*T*_min_, *T*_max_]. The free energy differences Δ*F*(*T*_*j*_) at each temperature point and the bias potential *V*_*n*_^MT^(*U*) are iteratively updated. At step *n*, they read
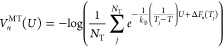
2
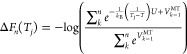
3where *T* indicates the temperature of the simulation thermostat, and *N*_T_ indicates the number of intermediate temperatures within the chosen temperature range. The values of the intermediate temperatures and *N*_T_ can be either automatically determined by OPES MultiThermal or explicitly set by the user.^29^

The presence of OPES MultiThermal in the SWISH-X strategy is particularly useful for accelerating the sampling of those cryptic pockets whose complex dynamics is associated with relatively high free energy barriers and cannot be fully captured by regular SWISH simulations. Furthermore, SWISH-X has no additional computational cost when compared to SWISH. Determining the optimal temperature range is a crucial parameter to fine-tune the SWISH-X strategy. It is important to ensure that the range is broad enough to allow significant sampling without triggering transitions to higher-energy unfolded states.

### 2.2 Simulation Protocols

#### Unbiased MD

All atomistic unbiased molecular dynamics simulations were performed using the GROMACS 2021.3 software package^40^ with the DES-Amber force field^41^ in combination with the TIP4P-D water model.^42^ The systems were equilibrated in three steps; refer to Supplementary Section 1.4 for further details. The final structure obtained from the equilibration process served as the initial configuration for the MD simulations. All systems were simulated in the NPT ensemble with periodic boundary conditions, using the same parameters as in the last equilibration step. The particle mesh Ewald method was used to account for long-range electrostatics, with a cutoff of 12 Å.^43^ A time step of 2 fs was used for all simulations after constraining the hydrogen stretching modes using the LINCS algorithm.^44^ The simulation times for each system are given in Table S1. The cumulative simulation time for all of the systems presented in this work is 36.5 μs.

#### Mixed-Solvent MD

All mixed-solvent simulations were run with GROMACS 2022.3 patched with Plumed 2.9.0.^40,45^ DES-Amber^41^ and TIP4P-D^42^ were selected as force field and water model, respectively. Benzene was used as probe molecule, and the same concentration (1 M) was used for all the presented systems. The choice of benzene was guided both by the mainly hydrophobic character of the PPI interfaces and by our previous tests of the performance of various mixed solvents in revealing different cryptic pockets.^24^ The benzene molecules used in the mixed-solvent simulations were parametrized using Gaussian 16^46^ with the Amber GAFF-2 force field^47^ and RESP charges. An interprobe repulsive potential was also applied to prevent phase separation.^18^ A link to the benzene parameter files used is available on GitHub; refer to the Data Availability section. To avoid potential protein unfolding due to the high concentration of probes, a contact map-based restraint was applied during the simulation. The optimal upper wall value for the contact map was determined by examining the fluctuations of the chosen contacts observed during unbiased simulations. The simulation parameters and equilibration protocol were consistent with those used in the unbiased MD simulations. The benzene molecules and the protein system were assigned to the same temperature coupling group. The simulation time for each system is given in Table S1.

#### SWISH

In this study, all SWISH simulations followed a standardized protocol. Specifically, we ran six parallel replicas for each system, with each replica having a different value of the scaling factor (λ). The scaling factors were uniformly distributed and ranged from 1.00 (nonscaled replica) to 1.35. Benzene was included in the simulations as a cosolvent at a concentration of 1 M. The benzene molecules and the protein system were assigned to the same temperature coupling group. The simulation parameters, with the exception of scaling factors, were consistent with those used in the unbiased MD simulations. Prior to each production run, six independent equilibration runs were performed, one for each λ value. We used the same equilibration protocol as that described for the unbiased MD simulations. Following the same approach used in the mixed-solvent simulations, we included a contact map-based restraint to prevent potential unfolding of the protein in replicas with high scaling factors. The optimal upper wall value for the contact map was determined by analyzing the fluctuations in the selected contacts observed during the unbiased simulations. We used the same benzene parameters as those used in the mixed solvent MD simulations. A link to a detailed guide on how to set up a SWISH simulation is available in the Data Availability section. All SWISH simulations were run with GROMACS 2021.3 patched with Plumed 2.8.0,^40,45^ the DES-Amber force field,^41^ and TIP4P-D water model.^42^ Additional information about the parameters of the various SWISH simulations presented can be found in Table S2.

#### SWISH-X

The SWISH-X simulations in this paper follow the same setup as that of the SWISH simulations presented above. Specifically, we employed the same number of replicas, scaling factors, contact map upper wall, benzene concentration, and MD setup for each system here presented. However, in contrast to SWISH, our SWISH-X protocol includes an OPES MultiThermal component in all six replicas. The selected temperature range went from the chosen thermostat temperature, 300 K, to 350 K for all systems except for HPV-11 E2, where the highest temperature was set to 330 K. We followed the same equilibration protocol as the one used for the SWISH simulations, allowing each replica to equilibrate at the corresponding λ-biased ensemble. All SWISH-X simulations were run with GROMACS 2022.3 patched with Plumed 2.9.0.^40,45^ A link to a detailed guide on how to set up a SWISH-X simulation is available in the Data Availability section. Additional information about the parameters of the various SWISH-X simulations presented can be found in Table S2. We provide an example of a successful potential energy exploration using SWISH-X (via the OPES MultiThermal bias) in Figure S1.

### 2.3 Dimensionality Reduction and Clustering

We devised a clustering protocol based on Principal Component Analysis (PCA),^31,32^ t-Distributed Stochastic Neighbor Embedding (t-SNE),^33^ and Hierarchical Density-Based Spatial Clustering of Applications with Noise (HDBSCAN).^34^ We defined all contacts between the center of mass of each residue within 7 Å of the center of the cryptic pocket in the corresponding protein reference structure and tracked their values in each Frame of a given simulation. Contacts were normalized using a sigmoid function (*S*) as follows
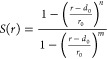
4where *r* denotes the distance between the center of mass of two residues, *r*_0_ was set to 0.8 nm, while *n* and *m* were set to 4 and 8, respectively.

Thus, for a given simulation of *m* frames, we obtained a matrix of size *m* × *n*, where each element represents a normalized value between 0 and 1 corresponding to the *n*-th contact. We performed a PCA on this matrix, keeping the first 50 components (obtaining an *m* × 50 matrix) before generating the t-SNE embedding. Since t-SNE is sensitive to the local environment and the data points on which it is trained, we applied both the PCA and t-SNE transformations to the structures obtained from open-like and either apo-unbiased, mixed-solvent MD, SWISH-X or SWISH simulations in order to have a reference for the open state in t-SNE space.

Specifically, we applied t-SNE in such a way that it models each high-dimensional (50 PCA dimensions) data point by a two-dimensional point so that similar structures are modeled by nearby points and dissimilar structures are modeled by distant points with high probability. The holo-like structures of the cryptic site were obtained from the unbiased MD runs starting from the holo structure of the main cryptic pocket of the protein under study. The ligands occupying the cryptic sites in the holo structures were removed before the simulations. The rationale for this choice is that it should capture not only the initial fully open conformation of the pockets but also possible semiopen conformations sampled during the simulation. We have verified that rerunning the analysis on the ligand-bound structures leads to a similar description (Supplementary Section 2 and Figure S2).

The structures resulting from the open-like simulations should resemble more open-like configurations of the cryptic site, provided that the pocket remains stable and does not close during the simulation. We verified this assumption for each system by monitoring the pocket volume during the unbiased holo-like simulations and selected only trajectories sampling open-like configurations of the cryptic pocket. This was not possible for IL-2, but we still included it in the analysis. The various t-SNE embeddings were obtained with the TNSE package from scikit-learn version 1.2.2.^48^ We used the default scikit-learn parameters for all of the embeddings.

We then clustered the various t-SNE projections with HDBSCAN, obtaining separate clusters that generally correspond to different configurations of the cryptic pocket. The HDBSCAN algorithm interprets clusters as regions of increased data density separated by regions of lower density or noise. In the HDBSCAN framework, a cluster consists of core samples that are in close proximity to each other and noncore samples that are in the vicinity of a core sample located at the edge of the cluster.

HDBSCAN’s clustering relies on the interpretation of data density. Specifically, density is defined primarily by two parameters: epsilon, a metric for measuring neighborhood distances, and the minimum number of neighboring data points, within an epsilon distance, required to designate a point as a core sample. HDBSCAN performs a series of DBSCAN^49^ runs using different epsilons and consolidates the results to identify a clustering configuration that optimizes stability across epsilon values. This approach allows HDBSCAN to detect clusters of different densities, distinguishing it from DBSCAN and making it more robust to parameter selection.

We tuned the minimum samples parameter to capture high-density areas in the t-SNE space by testing different values of this parameter. The minimum number of points in a neighborhood for that area to be considered a cluster, namely, the minimum cluster size, ranges from 200 to 75 for the systems analyzed. In the clustering procedure, we only considered data points from either apo-unbiased, mixed-solvent, SWISH-X or SWISH simulations, excluding data points from the holo-like unbiased simulations. Ten sample structures from every cluster in each obtained cluster map can be accessed as a PyMOL session via a link in the Data Availability section.

## 3 Results

### 3.1 TEM-1 β-Lactamase

**Figure 2:**
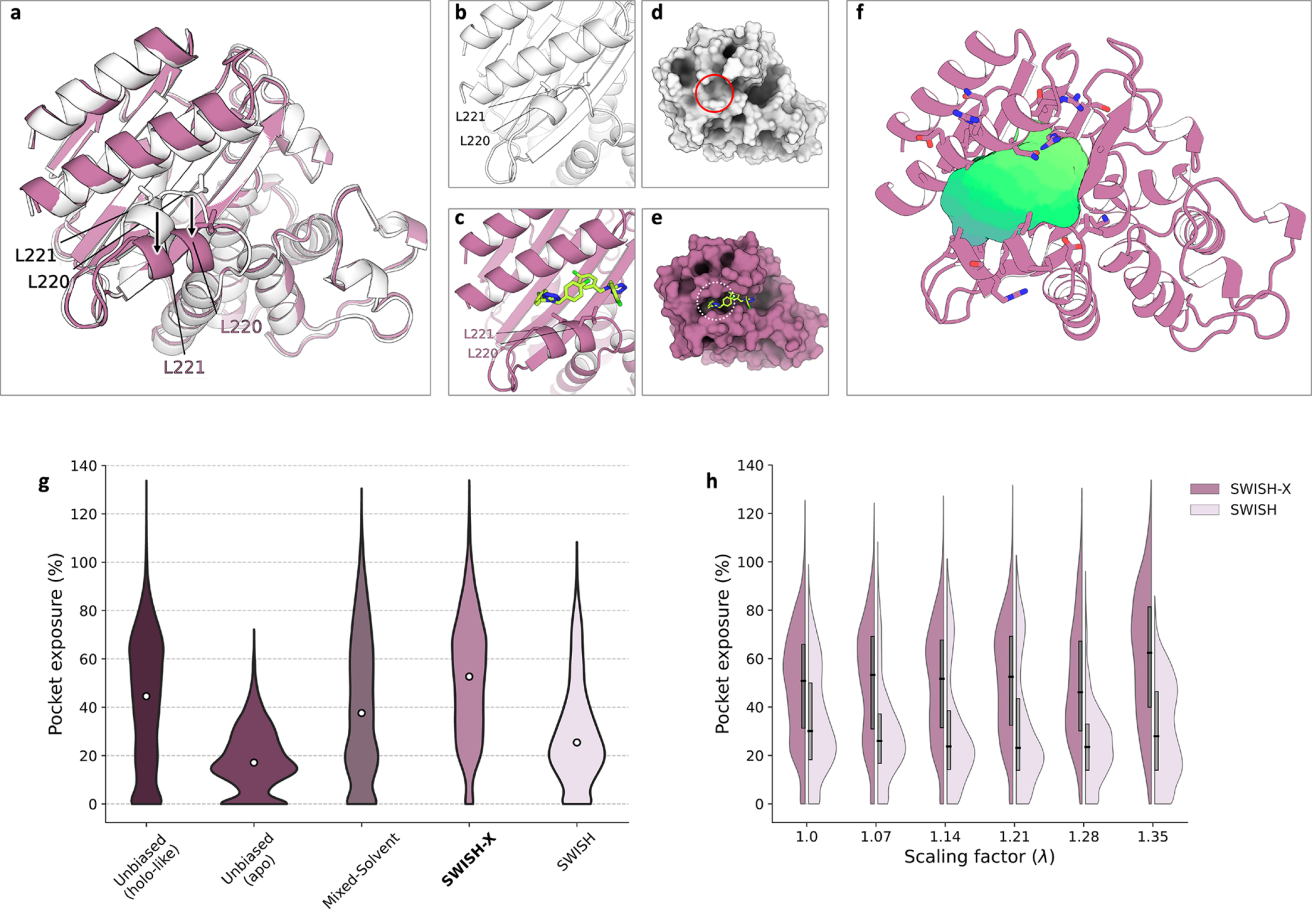
Structural description and sampling efficiency of TEM-1’s cryptic binding pocket. **a)** Structural alignment of TEM-1’s apo (white, PDB ID: 1JWP) and holo (pink, PDB ID: 1PZO) structures. Relevant residues are depicted as sticks and labeled, and ligands are omitted for clarity. **b**-**c**) Close-up views of the cryptic cavity in the apo and holo structures, respectively. Ligands are shown as green sticks. **d**-**e**) Surface representations of apo (white) or holo (pink) TEM-1. The cryptic pocket is not detectable in the apo crystal (solid red circle in panel d) but is visible in the ligand-bound state (dotted white circle in panel e). **f**) Location of the target cryptic pocket (green surface) within TEM-1’s structure, depicted as cartoon. **g**) Violin plots of the exposure of the selected cryptic pocket along different simulations. Pocket exposure is expressed as a percentage of the volume of the cryptic site (Å^3^) with respect to the volume of the corresponding pocket in the holo crystal (PDB ID: 1PZO). The simulations are presented as follows (left to right): holo-like unbiased MD, apo unbiased MD, mixed-solvent MD, SWISH-X, and SWISH. Exposure values of single replicas of a given simulation are merged into a unique violin plot. **h**) Comparison of the opening profiles along different replicas of TEM-1’s SWISH-X and SWISH simulations, respectively.

TEM-1 β-lactamase harbors two distinct cryptic pockets, one sandwiched between two alpha helices and a second one linked to the dynamics of the enzyme’s Ω-loop (Figure 2).^50^ To date, no experimental holo structure is available for the Ω-loop cryptic site. Therefore, we focused our analysis solely on the main cryptic cavity of the enzyme.

We have previously shown that SWISH, in combination with a 1 M concentration of benzene cosolvent molecules, successfully samples the opening of the largest cryptic cavity of TEM-1 β-lactamase.^24^ In this study, we ran a new SWISH simulation and three independent mixed-solvent MD runs starting from a closed configuration of the cryptic pocket (PDB ID: 1JWP) and compared the results obtained with the SWISH-X simulations. To evaluate the stability of the main cryptic cavity of TEM-1 beta-lactamase, we performed three unbiased simulations of the open-like state of the cryptic site. The open conformation was obtained by removing the ligand structures from the holo crystal structure of TEM-1 (PDB ID: 1PZO).

The change in the volume of the main cryptic site is shown in Figure 2g. Only one of the three unbiased simulations starting from the open-like configuration (unliganded PDB ID: 1PZO) predominantly explored open configurations of the cryptic site, with a median pocket exposure of 64%. In contrast, the other two unbiased simulations mostly sampled semiopen or closed states of the cryptic site (Figure S3). The control unbiased simulation of the apo state (PDB ID: 1JWP) in water confirmed the stability of the closed state, as also indicated by the time profile of the pocket’s exposure (Figure S3).

Similarly, our mixed-solvent MD simulations predominantly sampled closed-like and semiopen configurations of the pocket during most of the simulations (median exposure of 37.6%), with holo-like configurations becoming more prevalent at longer simulation times. The sampling obtained with SWISH indicates a limited exploration of the pocket’s open state, with a median site exposure of 25.5%. The shorter sampling times explain, at least in part, the more limited cavity opening observed compared to our previous study.^24^ In contrast, the SWISH-X simulation is characterized by a higher median exposure (52.7%), which is similar to the value obtained from the holo-like unbiased simulation that mostly explored open configurations of the cryptic site (64%, Figure S3).

When comparing the individual SWISH and SWISH-X replicas (Figure 2h), the SWISH-X simulation consistently displays a significantly higher volume of the cryptic pocket in all but one replica, suggesting a better sampling of its open conformation. This further indicates how a single SWISH-X simulation, with 250 ns per replica, is sufficient to capture the significant conformational changes associated with the opening of the cryptic site.

The significant difference in the sampling efficiency between SWISH and SWISH-X prompted us to further investigate the role of the temperature in aiding the opening of TEM-1’s main cryptic site. We therefore tested two additional OPES MultiThermal tempering schemes (Supplementary Section 3). The results show that allowing each replica to sample the same temperature range of 300–350 K gives the best results. Instead, if the temperature range sampled by each replica is either incrementally increased, forcing lower replicas to sample a lower temperature range, or globally broadened toward lower temperature values (from 300 to 350 K to 280–350 K), the median pocket exposure decreases to 33% and 38%, respectively (Figure S4).

**Figure 3:**
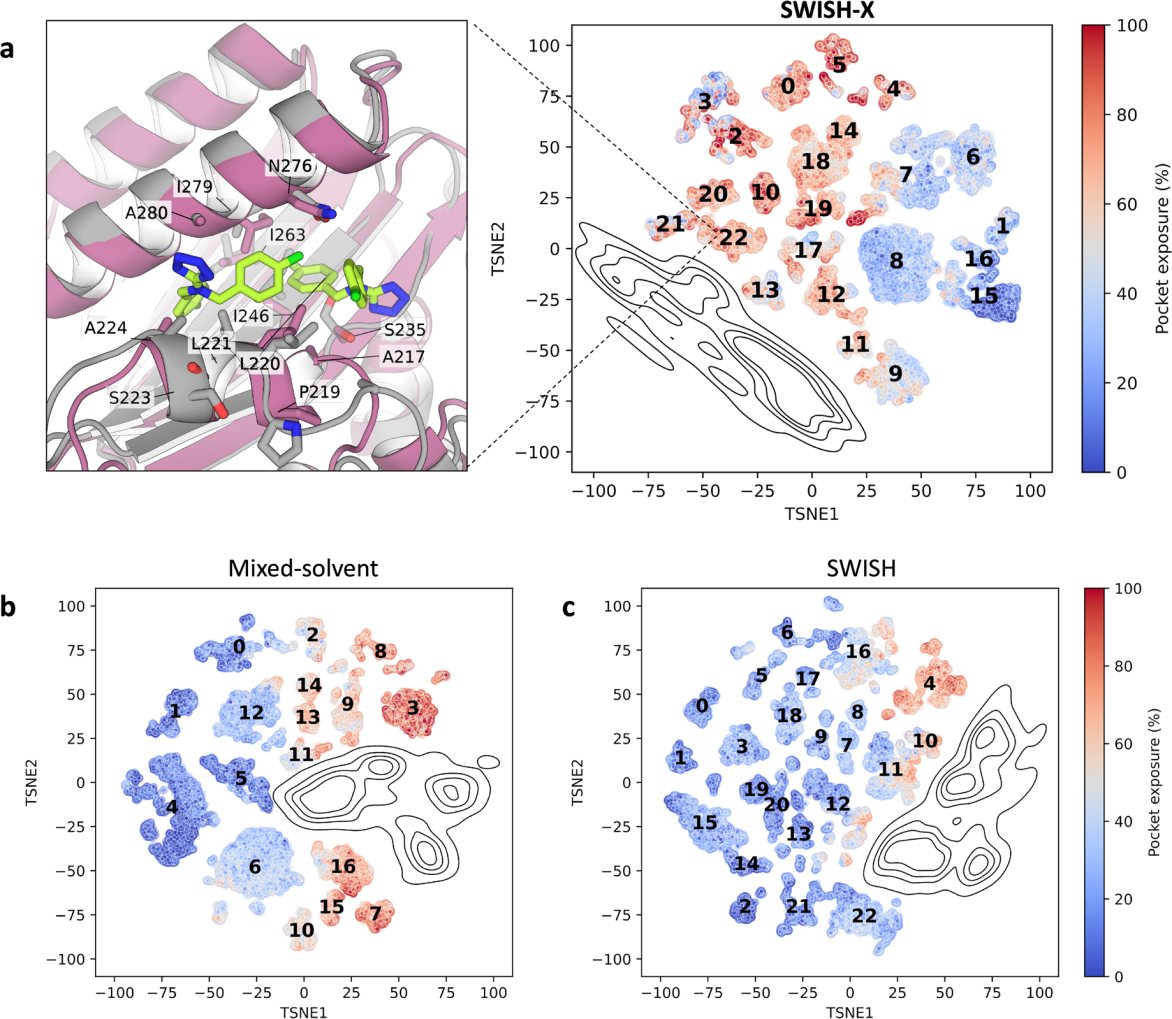
t-SNE cluster maps of TEM-1’s cryptic pocket. Each point in a given t-SNE space corresponds to a pocket configuration sampled during either a SWISH-X (**a**), mixed-solvent (**b**), or SWISH (**c**) simulation, with colors indicating the percentage of pocket exposure. Different numbers identify distinct clusters within each t-SNE cluster map. Isocontour lines highlight the region of the t-SNE space explored by holo-like simulations. In panel (**a**), the left-hand side displays the structural alignment of the cryptic site in the holo structure (gray, PDB ID: 1PZO) and in a representative structure extracted from the centroid region of cluster 22. The heavy atom RMSD of the alignment is 2.4 Å. Relevant pocket lining residues are shown as sticks and labeled. Ligands in the holo crystal are shown as sticks.

To better investigate the different conformations sampled during the various simulations, we performed a cluster map analysis on all of the resulting trajectories. We identified several clusters corresponding to different configurations of the cryptic pocket’s opening state. The results of the apo unbiased, mixed-solvent MD, SWISH-X, and SWISH simulations are shown in Figure 3 and Figure S5a.

In the SWISH-X simulation, clusters near the contour of the open-like configurations typically resemble open configurations of the cryptic pocket. This observation is further supported by the average percentage of exposure of the cryptic site for the structures in these clusters (Figure 3a). Additionally, when the t-SNE cluster map is colored by RMSD values with respect to the holo crystal, multiple clusters characterized by both high pocket exposure values and low RMSD values can be identified (clusters 10, 14, 18, 19, and 22 in Figure 3a and Figure S6c). These clusters correspond to crystal-like configurations sampled during the SWISH-X simulation.

In contrast, distant clusters generally correspond to different configurations of the pocket, mostly closed ones, thus being associated with higher RMSD values. Similarly, the mixed-solvent simulations successfully sampled the open configuration of the enzyme’s cryptic pocket (Figure 3b and Figure S6b). Although the cluster map of the SWISH simulation allowed us to identify open configurations of the cryptic site, these configurations were less populated than those obtained from the mixed-solvent and SWISH-X simulations (Figure 3).

Finally, we investigated how long the different simulation strategies take to sample an open crystal-like configuration of the cryptic pocket (Supplementary Section 4 and Table S4). Remarkably, SWISH-X sampled a crystal-like configuration of the enzyme’s cryptic site in less than 25 ns, whereas both our mixed-solvent and SWISH simulations achieved this much later, at 180 and 95 ns, respectively.

### 3.2 Bcl-X_*L*_

**Figure 4:**
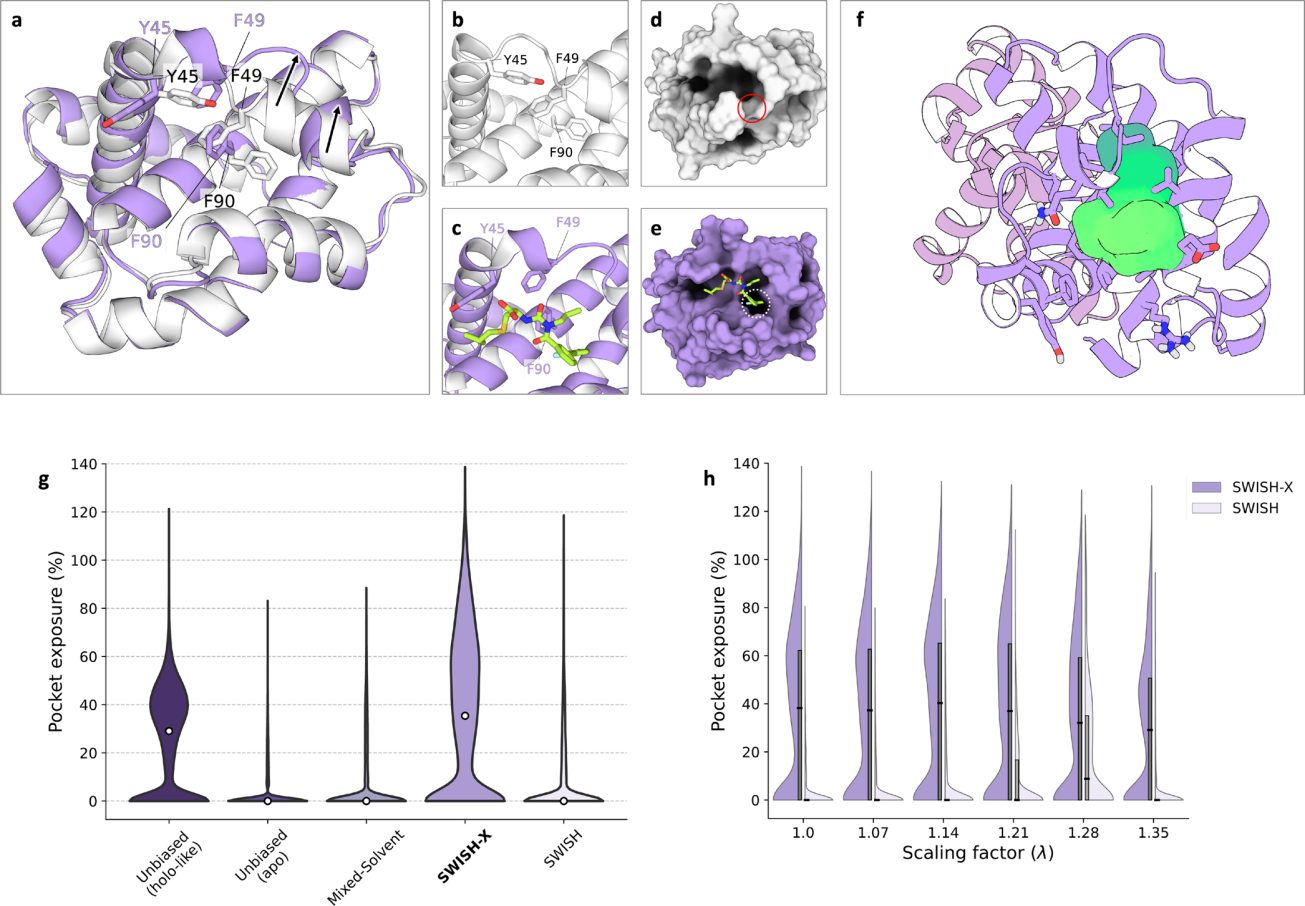
Structural description and sampling efficiency of Bcl-X_*L*_’s cryptic binding pocket. **a**) Structural alignment of Bcl-X_*L*_’s apo (white, PDB ID: 1R2D) and holo (violet, PDB ID: 4C52) structures. Relevant residues are depicted as sticks and labeled; inhibitor is omitted for clarity. **b**-**c**) Close-up views of the cryptic cavity in apo- and inhibitor-bound structures, respectively. Inhibitor is shown as green sticks. **d**-**e**) Surface representation of apo (white) or holo (violet) Bcl-4X_*L*_. The cryptic pocket is not detectable in the apo structure (solid red circle in panel d) but is visible in the inhibitor-bound state (dotted white circle in panel e). **f**) Location of the target cryptic pocket (green surface) within Bcl-X_*L*_’s structure, depicted as cartoon. **g**) Violin plots of the exposure of the selected cryptic pocket along different simulations. Pocket exposure is expressed as a percentage of the volume of the cryptic site (Å^3^) with respect to the volume of the corresponding pocket in the holo crystal (PDB ID: 4C52). The simulations are presented as follows (left to right): holo-like unbiased MD, apo unbiased MD, mixed-solvent MD, SWISH-X, SWISH. Exposure values of single replicas of a given simulation are merged into a unique violin plot. **h**) Comparison of the opening profiles along different replicas of Bcl-X_*L*_’s SWISH-X and SWISH simulations, respectively.

We prepared and simulated two different structures of Bcl-X_*L*_, an apo (PDB ID: 1R2D) and a holo-like conformation obtained from the inhibitor-bound crystal (PDB ID: 4C52). The superposition of the two states clearly shows how the binding of the small molecule exposes a cryptic pocket that is not detectable in the ligand-free state (Figure 4a-f). The exposure of the cryptic pocket requires distinct structural rearrangements within the binding region. These rearrangements include the movement and partial unfolding of a two-turn α-helix, along with the reorientation of residues Tyr45, Phe49, and Phe90 that together effectively reveal a deeper cavity.

We began by assessing the stability of the holo-like (PDB ID: 4C52) state of the cryptic pocket in its ligand-free configuration. The resulting pocket’s exposure profiles clearly indicate a metastable conformation of the cryptic site with a median exposure of 29% (Figure 4g and Figure S7). A careful examination of these trajectories revealed that the larger groove of the pocket remained stable and retained a configuration similar to that of the holo crystal, suggesting a high energy barrier associated with the movement of the α helix. In contrast, the deeper cavity of the pocket rapidly closed in all simulations due to the unfavorable, solvent-exposed conformation of Phe90 in the absence of the inhibitor.

To confirm that the closed conformation of the pocket is the most stable in the absence of the ligand, we conducted three independent unbiased simulations, each lasting 500 ns, starting from the apo conformation (PDB ID: 1R2D). As expected, none of the two conformational changes required to expose the cryptic pocket were sampled in these simulations, confirming the presence of a high energy barrier associated with the formation of the cavity (Figure 4g and Figure S7). Similarly, our three independent mixed-solvent MD runs, with a total cumulative simulation time of 1.5 μs, were unsuccessful in sampling the opening of the cryptic site. The obtained pocket exposure profiles are in line with those of the apo configuration in water, with the exception of one of the replicas (Figure S7). This specific replica, namely replica 1, partially sampled the movement of the α-helix necessary to reveal the cavity within 300 ns. However, we did not observe the flip of Phe90 required to expose the deeper subpocket.

Subsequently, we ran a SWISH simulation (6 replicas, 250 ns each) to attempt to recover the open state starting from a closed pocket conformation. The SWISH simulation only partially revealed Bcl-X_*L*_’s hidden cavity, as indicated by the obtained exposure profiles (Figure S7). Notably, scaling the protein–water interactions yielded significantly better results when compared with the unscaled mixed-solvent MD simulations. This can also be observed in the violin plots of the individual SWISH replicas (Figure 4h), which show how higher scaling factors aided the sampling of open-like configurations of the cryptic site.

We therefore tried to recover the open conformation of Bcl-X_*L*_’s cryptic pocket by running a SWISH-X simulation (1.5 μs of cumulative sampling time). The results showed a significant improvement in the sampling of the cryptic cavity, with a median site exposure of 35.4%. The comparison of the volume profiles of each individual replica of both the SWISH and SWISH-X simulations highlights how SWISH-X effectively samples the opening of the cryptic site, whereas SWISH fails to do so (Figure 4h).

**Figure 5:**
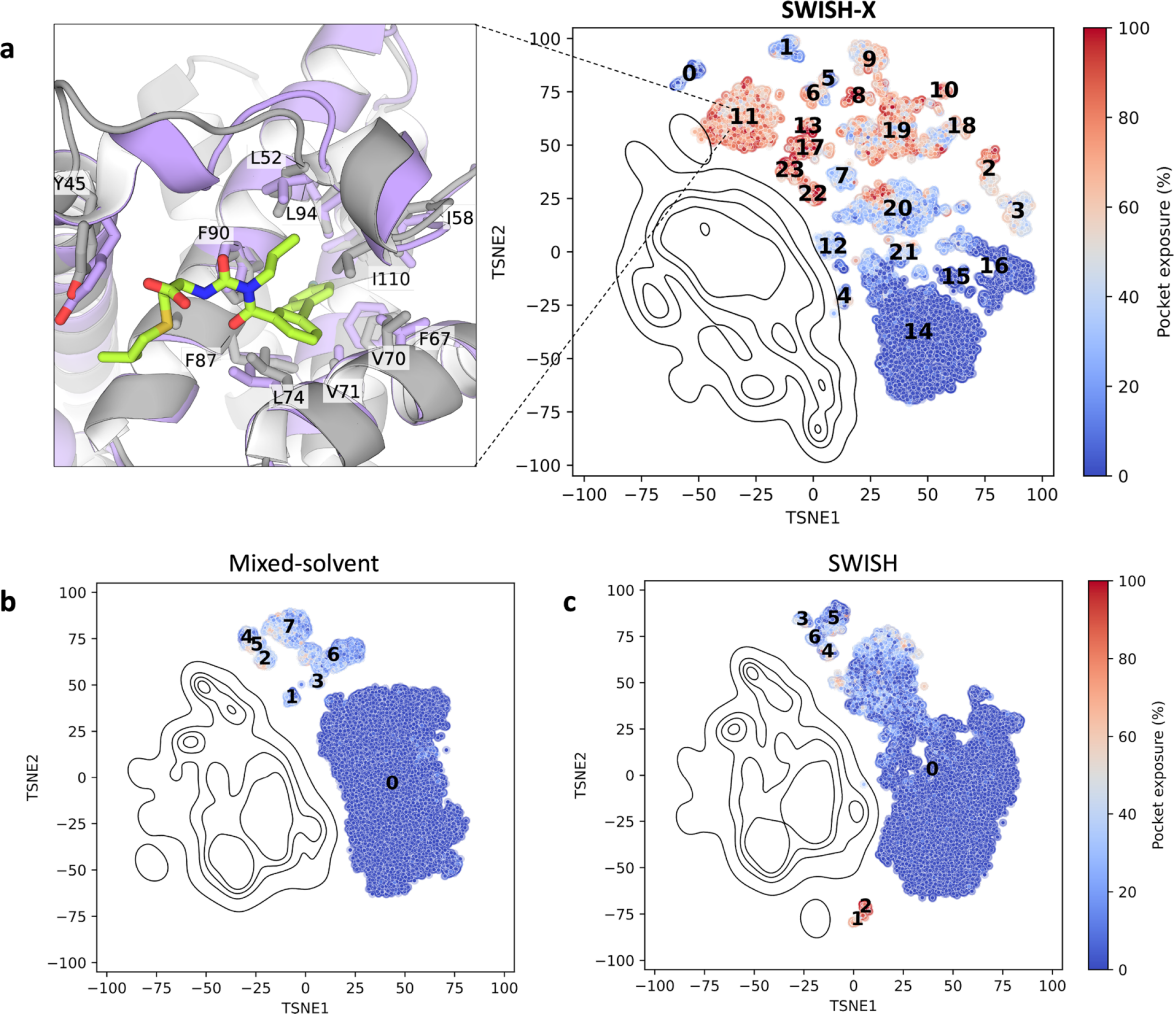
t-SNE cluster maps of Bcl-X_*L*_’s cryptic pocket. Each point in a given t-SNE space corresponds to a pocket configuration sampled during either a SWISH-X (**a**), mixed-solvent (**b**), or SWISH (**c**) simulation, with colors indicating the percentage of pocket exposure. Different numbers identify distinct clusters within each t-SNE cluster map. Isocontour lines highlight the region of the t-SNE space explored by holo-like simulations. In panel (**a**), the left-hand side displays the structural alignment of the cryptic site in the holo structure (gray, PDB ID: 4C52) and in a representative structure extracted from the centroid region of cluster 11. The heavy atom RMSD of the alignment is 1.3 Å. Relevant pocket lining residues are shown as sticks and labeled. Ligands in the holo crystal are depicted as sticks.

The cluster map analysis of all resulting trajectories confirms that only SWISH-X effectively samples holo-like states of the cryptic cavity (Figure 5 and Figure S5e). One cluster, in particular, explores a region of the conformational space similar to the one explored by the open-like simulations (Figure 5a). The median pocket exposure of this cluster, namely, cluster 11, is 69.6%. Moreover, when visualizing the same t-SNE cluster map by RMSD values, calculated with respect to the holo crystal, cluster 11 displays a median RMSD of 1.6 Å (Figure S6g). These observations indicate that cluster 11 is populated with structures that closely resemble the holo crystal structure.

A similar analysis of the configurations obtained from the mixed-solvent MD simulations confirms that the pocket’s opening is not sampled (Figure 5b). This is further supported by visualizing the RMSD values for each pocket configuration in the different cluster maps (Figure S6f). All clusters from our mixed-solvent simulations are characterized by high RMSD values and low pocket exposure values, consistent with the sampling of predominately apo-like configurations of the cryptic site. Similarly, the SWISH simulation mainly sampled closed configurations of Bcl-X_*L*_’s cryptic pocket (Figure 5c and Figure S6h). Nevertheless, it was possible to identify two clusters, namely, clusters 1 and 2, that have a median pocket exposure of 69.7% and 93.3% and a median RMSD value of 2.5 and 2.4 Å, respectively. While the structures belonging to these clusters can be considered similar to the holo crystal, their number is significantly lower compared to the ones in cluster 11 from our SWISH-X simulation.

The analysis of the opening times confirmed that SWISH-X and SWISH are the only two sampling strategies that successfully sample the full opening of Bcl-X_*L*_’s cryptic site (Supplementary Section 4 and Table S4). SWISH-X sampled holo-like states (with RMSD < 2 Å) with pocket exposure values of at least 60% and 80%, within 57 ns of simulation. To explore similar configurations of the pocket, SWISH required 227 ns of simulation. On the contrary, our mixed solvent simulations reach a crystal-like state with a pocket exposure of at least 60% in 214 ns but fail to sample a structure with an exposure of 80% and an RMSD < 2 Å.

### 3.3 Other PPI Sytems

### IL-2

**Figure 6:**
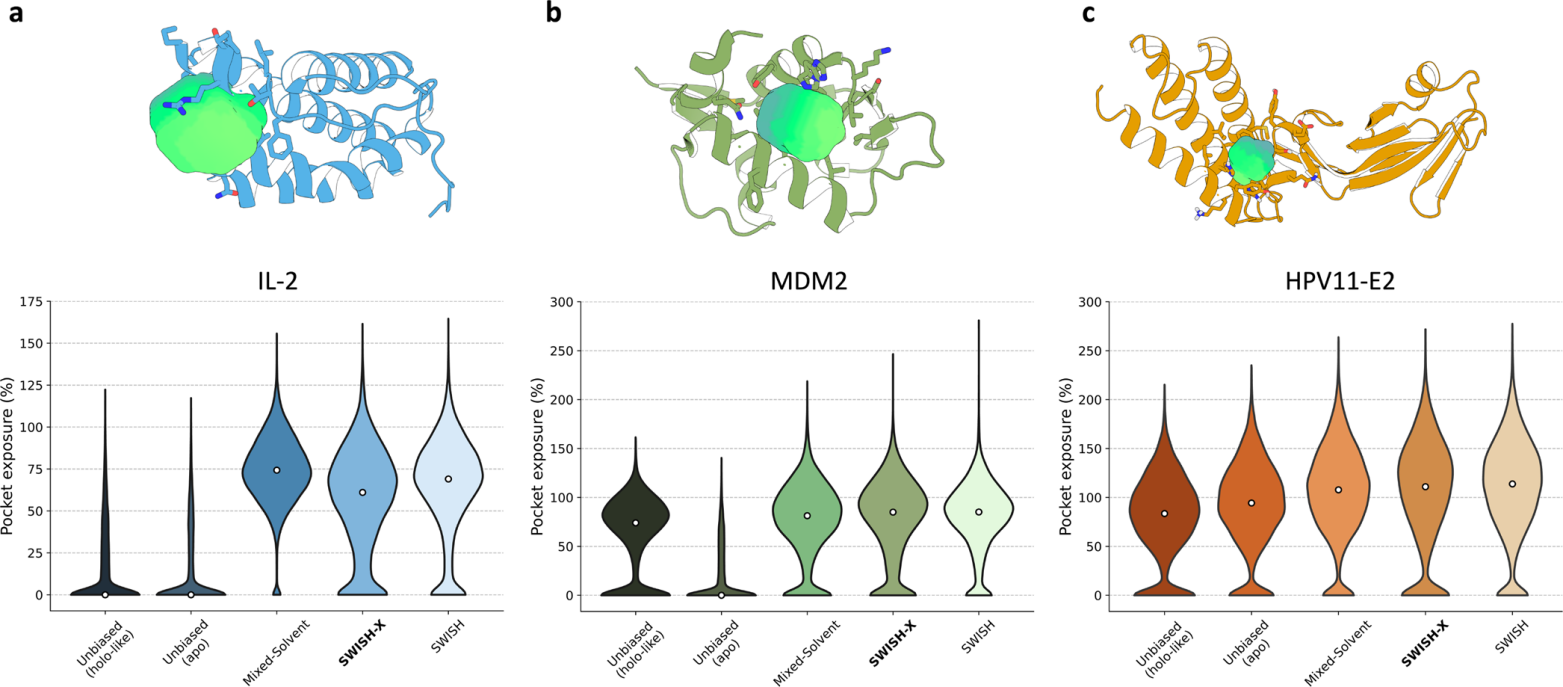
Cryptic pockets at different PPI interfaces. **a)***Top panel:* location of the target cryptic pocket (green surface) within inhibitor-bound IL-2’s structure (PDB ID: 1PY2). *Bottom panel:* violin plots of the exposure of the selected IL-2’s cryptic pocket along different simulations. **b**) *Top panel:* location of the target cryptic pocket (green surface) within inhibitor-bound MDM2’s structure (PDB ID: 5LAV). *Bottom panel:* violin plots of the exposure of the selected MDM2’s cryptic pocket along different simulations. **c**) *Top panel:* location of the target cryptic pocket (green surface) within inhibitor-bound HPV-11 E2’s structure (PDB ID: 1R6N). *Bottom panel:* violin plots of the exposure of the selected HPV-11 E2’s cryptic pocket along different simulations. Protein structures are depicted as cartoons. Relevant pockets lining residues are shown as sticks, and inhibitors are omitted for clarity (*top panels*). Pocket exposures are expressed as percentages of the volume of the cryptic site (Å^3^) with respect to the volume of the corresponding pocket in the holo crystal. The simulations are presented as follows (left to right): holo-like unbiased MD, apo unbiased MD, mixed-solvent MD, SWISH-X, and SWISH. Exposure values of single replicas of a given simulation are merged into a unique violin plot (*bottom panels*).

IL-2 is a well-characterized model system harboring a cryptic binding pocket at its PPi interface. The opening of the cryptic site reveals a binding groove that is not detectable in the apo IL-2 crystal (Figure S8a-e). In our MD simulations of apo IL-2, we mainly sampled closed conformations of the cryptic pocket (Figure 6a and Figure S9). Furthermore, simulations starting from unliganded open conformations obtained from the holo IL-2 crystal (PDB ID: 1PY2) also predominantly explored apo-like conformations, with the pocket promptly closing in all replicas (Figure 6a and Figure S9).

On the other hand, our mixed-solvent MD runs successfully captured the open conformation of the cryptic cavity with a median exposure of 74.3% (Figure 6a and Figure S9). These results are consistent with previous studies^20,21^ and confirm how mixed-solvent MD is effective in sampling superficial cryptic sites that do not require major conformational rearrangements to expose the cavity. As previously reported, SWISH can successfully sample the opening of the cryptic pocket of IL-2.^24^ This was further confirmed by our new SWISH simulation, resulting in a median pocket exposure of 69% (Figure 6a and Figure S9). The results are comparable to those obtained with the mixed-solvent MD simulations.

We ran a SWISH-X simulation to determine whether the temperature fluctuations could accelerate the sampling of this cryptic pocket or access different open-like states. Interestingly, SWISH-X sampled lower volume configurations of the cryptic site with a median exposure of 61% (Figure 6a and Figure S9). This is due to the fact that scaling the temperature not only favors open configurations of the pocket but also increases the transition rate between open and closed configurations. This effect is especially pronounced at lower scaling factors, where unscaled water molecules do not stabilize open cavity configurations, whereas higher scaling factors tend to favor and stabilize open configurations of the cryptic site.

We plotted the sampled configurations from the apo unbiased, mixed-solvent MD, SWISH and SWISH-X simulations in their respective t-SNE spaces (Figures S5–S6i-l). Both the apo- and holo-like unbiased simulations sampled the same region of configurational space, indicating a predominantly closed conformation of the pocket. The clusters obtained from the mixed-solvent MD, SWISH and SWISH-X trajectories are similar in all cases, with the majority of the structures corresponding to open-like configurations of the cryptic site. The characteristic cryptic site opening time was comparable in all the simulations we ran, with complete crystal like openings happening within 5 ns of each simulation (Supplementary Section 4 and Table S4).

### MDM2

We assessed the stability of the open state of the cryptic pocket using unbiased MD simulations of the holo-like structure (Figure S8f-j) in water, revealing a stable pocket with median exposure of 74% (Figure 6b). However, despite three 500 ns MD simulations starting from the closed conformation of the cryptic cavity (Figure S10), we did not observe a consistent opening of the cryptic pocket (Figure 6b and Figure S10).

We then performed mixed-solvent MD, SWISH and SWISH-X simulations to attempt to recover the open conformation of the cryptic pocket from its closed configuration. The results of the mixed-solvent simulations are consistent with a good sampling of MDM2’s cryptic site (median exposure of 81.3%, Figure 6b and Figure S10). These results are in good agreement with previous studies that also managed to expose this pocket with different probe molecules.^20,21^ In the case of SWISH and SWISH-X simulations, the structure of the protein unfolded at the highest lambda value (1.35). We therefore excluded this replica from the analyses. In the remaining five replicas of both SWISH and SWISH-X simulations, we observed a good sampling of the open conformation of the cryptic site, with exposure values of 85% and 85.1%, respectively (Figure 6b and Figure S10).

The results of the cluster map analysis confirmed our previous observations. The clusters obtained from the apo simulations mostly corresponded to closed conformations of the pocket (Figure S5m). In contrast, in the cluster maps obtained from mixed-solvent SWISH-X and SWISH simulations, we observed clusters primarily containing open-like configurations of the cryptic site (Figure S5n-p).

Notably, different open conformations of the original pocket were sampled during the SWISH-X simulation, as indicated by the RMSD values in the corresponding t-SNE space (Figures S5–S6o). We observed a number of states in which the protein displayed new cavities and exposed larger sites and tunnels originating from the original location of the cryptic site (Figure S11). This was also observed, to a lesser extent, in the SWISH simulation. Sampling higher temperatures plays an important role in accessing these states, although only experiment can verify whether they are an artifact of the simulations or actually physical and accessible states in the presence of the right ligand.

When comparing the opening times of MDM2’s cryptic site, SWISH-X was able to sample open, crystal-like conformations of the pocket within 2 ns of simulation (Supplementary Section 4 and Table S4). In contrast, mixed-solvent MD and SWISH sampled configurations similar to the holo crystal in 22 and 8 ns, respectively.

### HPV-11 E2

The opening of HPV-11 E2’s cryptic pocket is associated with the motion of the side chains of three residues, namely, Tyr19, His32, and Leu94, in the protein’s interdomain hinge region (Figure S8k-o). As the exposure of the pocket requires minor side chain rearrangements, the open state was extensively sampled also during all the unbiased simulations starting from the apo state (Figure 6c and Figure S12). Similar results were obtained for the holo-like simulations, indicating that the residues lining the cryptic pocket can sample both open and closed conformations during unbiased MD simulations, independently of the initial pocket state. These results suggest that the opening of this pocket is associated with a low energetic barrier and indicate how, in some cases, unbiased simulations in water are sufficient to reveal targetable cryptic cavities. This is consistent with previous findings suggesting that short MD simulations can reveal hidden binding sites.^35^

We ran exploratory mixed-solvent MD, SWISH and SWISH-X simulations to determine if we could sample different open conformations other than the crystallographic one. The resulting pocket exposure profiles showed a slightly higher median exposure along the mixed-solvent, SWISH and SWISH-X simulations compared to the apo- and holo-like simulations (Figure 6c and Figure S12). By analyzing the cluster maps, colored by RMSD and pocket exposure values, of the mixed-solvent MD, SWISH and SWISH-X simulations, we identified structures with larger pocket volume (Figures S5–S6r-t). This is likely due to the presence of stabilizing benzene cosolvent molecules during these simulations.

To a lesser extent, similarly large pocket volume values for the cryptic site were obtained in both holo-like and apo simulations. This indicates that unbiased MD is sufficient to successfully sample the full dynamics of HPV-11 E2’s cryptic site. Interestingly, for this relatively easy case, SWISH-X and mixed-solvent MD provided comparable sampling times of holo-like configurations of HPV-11 E2’s cryptic site, achieving site exposure in 4 and 5.2 ns, respectively (Supplementary Section 4 and Figure S4).

## 4 Discussion

We investigated the dynamics associated with the opening of druggable cryptic cavities at protein–protein interfaces in four challenging systems as well as in the single protein TEM-1 β-lactamase. Our results show that SWISH-X effectively exposes the cryptic pockets of all systems presented and offers a significant speedup compared to the other methods tested, especially for more challenging cases such as TEM-1 β-lactamase and Bcl-X_*L*_.

In agreement with previous studies,^21,51,52^ mixed-solvent simulations proved to be effective in revealing superficial cryptic cavities, where the opening is primarily characterized by loop movements and side chain motions. On the other hand, in complex systems such as TEM-1 β-lactamase, mixed-solvent MD simulations only partially succeed in sampling the structural rearrangements associated with the opening of the cryptic cavity. SWISH, used in combination with probe molecules, requires longer sampling times to effectively explore the helix displacement necessary to reveal the cryptic site of the enzyme.

Moreover, in systems characterized by complex conformational changes and higher energy barriers, both mixed-solvent MD and SWISH fail to fully sample the opening of the cryptic cavity. In the case of Bcl-X_*L*_, only the SWISH-X simulation accurately captures the opening of the cryptic site, successfully sampling the backbone motion and side chain reorientation required to expose the cavity. Additionally, the results obtained for TEM-1 β-lactamase show that SWISH-X not only successfully exposes the enzyme’s cryptic pocket but does so in significantly less simulation time compared to SWISH and mixed-solvent MD.

In general, mixed-solvent MD simulations should be effective in revealing cryptic sites for systems where the opening of the cavity is primarily governed by an induced-fit mechanism or a conformational selection mechanism associated with a low energy barrier. However, mixed-solvent MD is not expected to work as well in systems where the formation of the cryptic site follows a conformational selection mechanism characterized by a substantial energy barrier. In these cases, the conformational change becomes the time-limiting step that dictates the opening of the pocket. Simulation methods that do not accelerate the crossing of such kinetic barriers fail to successfully sample the transitions needed to open the cryptic pocket within a reasonable simulation time. On the other hand, methods such as SWISH-X, which lower kinetic barriers independently of the underlying mechanism, successfully accelerate the sampling of such conformational changes.

Interestingly, in the context of PPIs, conformational selection appears to play an important role in revealing interface binding sites.^53^ This further supports the use of SWISH-X as an effective tool for discovering novel cryptic sites at the PPI interfaces. Additionally, when searching for cryptic sites at PPI interfaces, SWISH-X is completely interface-agnostic. This means that our approach can be easily applied to individual partner proteins, eliminating the need for a structure of the PPI complex. However, if the interaction interface is known, then this additional information can be used to enhance the conformational sampling of the interface region by specifically scaling the water interactions with the residues at the PPI interface or by enhancing the sampling of specific degrees of freedom.

Furthermore, in the case of cryptic pockets, where both induced-fit and conformational selection mechanisms play a role in their formation, the combination of SWISH-X with various mixed solvents should provide a powerful strategy for revealing the cavities.

As mentioned above, the exploratory capacity of SWISH-X must be controlled to avoid partial or complete unfolding of the target protein. In this respect, the choice of molecular probes, their concentration, the temperature range, and the λ window play an important role. Regarding the temperature range, our tests indicate that 300–350 K should work in most systems. In terms of probes, benzene tends to expose most of the hydrophobic cavities. For other pockets, different probes will be more efficient, as has been shown extensively in the mixed solvent simulation literature.^54,55^ Finally, it is advisible to use a restraint (upper wall) on the contact map of the protein.

We conclude that for the detection of novel cryptic pockets, both on single proteins and at PPI interfaces, especially when little is known about their structure and energetics, the use of a combined and generally applicable method such as SWISH-X might be very beneficial.

Finally, we provide analysis tools to generate structure-based cluster maps from the simulations. The cluster maps capture different pocket configurations and identify holo-like structures along trajectories that start from an apo state of the target protein. The insights gained from these cluster maps should facilitate the use of the simulation data in subsequent virtual screening pipelines for drug discovery.

## Data Availability

SWISH-X is implemented in PLUMED,^45^ from version 2.8 onward, in combination with GROMACS.^40^ The input files to replicate all the simulations are available on PLUMED NEST.^56^ SWISH-X tutorial: https://github.com/Gervasiolab/Gervasio-Protein-Dynamics/tree/master/swishX_bootcamp. The benzene parameters can be found at: https://github.com/Gervasiolab/Gervasio-Protein-Dynamics/tree/master/swishX_bootcamp/fragments/. SWISH tutorial: https://github.com/Gervasiolab/Gervasio-Protein-Dynamics/tree/master/swish_bootcamp. The presented cryptic pockets are available at: https://github.com/Gervasiolab/Gervasio-Protein-Dynamics/tree/master/swish_expanded/cryptic_pockets. Example structures from each t-SNE cluster map are available at: https://github.com/Gervasiolab/Gervasio-Protein-Dynamics/tree/master/swish_expanded/cluster_maps_structures. All data supporting the findings of this study are available on request. Example Jupyter Notebooks employed for the analysis of the presented data are available at: https://github.com/Gervasiolab/Gervasio-Protein-Dynamics/tree/master/swish_expanded/.

## Supporting Information Available

Example of potential energy fluctuations during a SWISH-X simulation; details on system selection, preparation and pocket detection; further details on the simulation protocol; impact of the initial data on t-SNE embeddings; additional information on cumulative simulation times for each system; some enhanced sampling parameters used in the simulations; exposure profiles of the various cryptic pockets along different simulations; effect of different tempering schemes on SWISH-X simulations; t-SNE RMSD and volume maps of the different cryptic pockets; opening times of cryptic sites under various simulation strategies; additional structural information on some of the cryptic pockets presented (PDF)
