# Supplementary material for: SWISH-X, an Expanded Approach to Detect Cryptic Pockets in Proteins and at Protein–Protein Interfaces
Source: J Chem Theory Comput. 2024 Apr 2;20(8):3335–48. doi: 10.1021/acs.jctc.3c01318 (PMC11044271; doi:10.1021/acs.jctc.3c01318)
Supplement: Supplementary file 1 — ct3c01318_si_001.pdf [file ct3c01318_si_001.pdf]

Supplementary Information

SWISH-X, an Expanded Approach to Detect Cryptic Pockets in Proteins and at Protein-Protein Interfaces

Alberto Borsatto,[†,‡,¶] Eleonora Gianquinto,[§] Valerio Rizzi,[†,‡,¶] and Francesco Luigi Gervasio[*,†,‡,¶,‖,⊥]

†*School of Pharmaceutical Sciences, University of Geneva, 1205 Geneva, Switzerland*

‡*Institute of Pharmaceutical Sciences of Western Switzerland, University of Geneva, 1205 Geneva, Switzerland*

¶*Swiss Institute of Bioinformatics, 1015 Lausanne, Switzerland*

§*Department of Drug Science and Technology, University of Turin, 10125 Turin, Italy*

‖*Department of Chemistry, University College London, WC1H0AJ London, United Kingdom*

⊥*Institute of Structural and Molecular Biology, University College London, WC1E7JE London, United Kingdom*

E-mail: francesco.gervasio@unige.ch

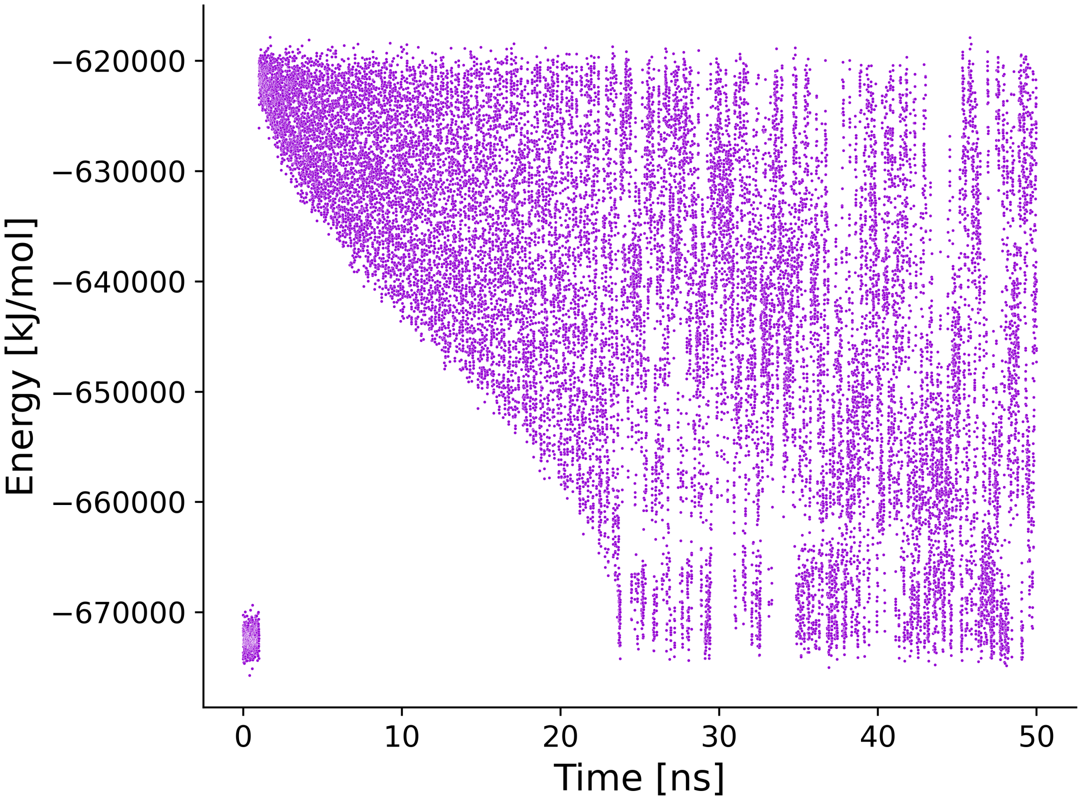

Figure S1: **Example of the potential energy fluctuations during a SWISH-X simulation.** Potential energy values of an example system along the first 50 ns of a SWISH-X replica. At the start of the simulation, the potential energy of the system is governed solely by the thermostat (300 K). In this example, after 1 ns, OPES MultiThermal introduces a bias on the potential energy that drives the system to match the temperature range selected (300-350 K). At convergence, the potential energy should fluctuate evenly across its range, as shown here from approximately 25 ns.

1 Methods

1.1 Selection of PPIs

A brief description of the four target proteins harbouring cryptic pockets at their protein-protein interfaces follows.

Bcl-X$_L$. B-cell lymphoma-extra-large (Bcl-X$_L$), a member of the Bcl-2 protein family, is a pro-survival mitochondrial transmembrane protein. However, its interaction with BCL2 Antagonist/Killer 1 (Bak) protein activates the mitochondrial apoptotic pathway and mitophagy.[1] Consequently, Bcl-X$_L$ has become a target for anticancer agents such as venetoclax and navitoclax. These agents mimic the BH3 domain of Bak protein and promote the apoptotic cascade. Bcl-X$_L$ (UniProt: Q07817, sequence: 1-MSQ...LYG-196) is assumed to form a homodimer in its apo form with a N-swapped domain(PDB ID: 1R2D).[2] In its holo form, each of the two protomers binds to either BH3 or a BH3-mimicking inhibitor (PDB ID: 4C52)[3], resulting in a heterotetramer assembly.

IL-2. Interleukin-2 (IL-2) is a single-chain soluble cytokine hormone released by T-cells, which binds to IL-2 receptors (IL-2R) to modulate the normal immune response.[4] The IL-2R family includes three monomeric transmembrane proteins, IL-2Rα, IL-2Rβ, IL-2Rγ. While IL-2Rα firstly binds and concentrates IL-2 on the T-cell surface, the subsequent binding to IL-2Rβ and IL-2Rγ activates the intracellular response, leading to T-cell growth and differentiation. While agonists of the IL-2/IL-2Rα PPI (IL-2-like peptides) have been developed for anticancer immunotherapy, antagonists have been pursued as a promising strategy to mitigate inflammatory and autoimmune diseases. One of the main challenges in developing small-molecule antagonists of the IL-2/IL-2Rα PPI is to target a large and discontinuous epitope. Nonetheless, a first hit compound Ro26-4550 with low micromolar activity was discovered,[5] and later further optimised to a nanomolar inhibitor by fragment-based ap-

proaches, proving the druggability of the IL-2/IL-2Rα PPI.[6]

MDM2. Murine double minute 2 (MDM2) protein is an E3 ligase that inhibits the function of the transcription factor p53 by binding to p53's transactivation domain. This interaction promotes the export of p53 outside of the nucleus and eventually leads to p53 ubiquitylation and degradation by the proteasome. Notably, MDM2 is overexpressed in 7% of various tumour types. Targeting the MDM2/p53 interaction has been actively pursued as a strategy to reactivate p53 and inhibit tumor growth. There are currently nine inhibitors being evaluated in clinical trials.[7] These inhibitors mimic the key interactions between MDM2 and p53, primarily involving three hydrophobic residues on p53: Phe19, Trp23, and Leu26. MDM2's surface accommodates these residues in three distinct sub-sites, created by the displacement of residues Phe86, His96, Val93, and Tyr100. For our simulations, we selected an NMR structure of the apo form of MDM2 (PDB ID: 1Z1M) and the X-ray structure of MDM2 bound to the 6b inhibitor (IC_{50} MDM2/p53 = 0.819 μM, PDB ID: 5LAV), as reported by Gollner and colleagues.[8]

HPV-11 E2. The E2 protein from Human Papilloma Virus type 11 (HPV-11) is a replication initiation factor that recruits the E1 helicase to improve the DNA binding activity. Biological assembly of E2 is dimeric and is composed of a C-terminal DNA binding/dimerization domain connected to the N-terminal transactivation domain (TAD) by a hinge region. The TAD domain is responsible for the binding to E1 helicase and shows a helix bundle and antiparallel beta sheet. To date, only two structures of the HPV-11 E2 TAD have been resolved (PDB IDs: 1R6K, 1R6N), the first corresponding to the apo conformation and the second bound to two spirocyclic indandione inhibitors.[9] The ligand pocket is not visible in the apo state of HPV-11-E2 (PDB ID: 1R6K) but becomes apparent upon binding of inhibitors (PDB ID: 1R6N).

1.2 Protein preparation

All systems were prepared following the same protocol. The structures of the selected proteins were downloaded from the RCSB PDB. For each protein, we selected a holo state (inhibitor-bound) and an apo state (Table 1). PDB structures were chosen taking into account the crystal resolution, the absence of mutations and the completeness of the sequence. Titratable residues were protonated at pH = 7.4 using the H++ webserver (http://newbiophysics.cs.vt.edu/H++/). All systems are functional as monomers, except for Bcl-X_L, which is functional as a homodimer. While the holo form of Bcl-X_L in PDB ID: 4C52 reports the correct dimeric assembly, the apo form of Bcl-X_L in PDB ID: 1R2D shows only one protomer. To recreate the correct dimeric assembly, after alignment of 1R2D to 4C52, the protomer in 1R2D was copied and aligned to chain B in PDB ID: 4C52. Finally, all truncated systems were capped at N- and C-termini with acetyl and N-methylammine groups, respectively.

1.3 Pocket detection

In order to assess the crypticity of the binding pockets within the studied systems and to quantify their physicochemical properties, we performed an analysis of the energy-minimised and protonated holo-crystal structures, as detailed in Table 1 in the main text, for each system. The ligands were removed from all holo structures prior to pocket detection.

The analysis was performed using Fpocket 4.0, a geometry-based cavity detection algorithm.[10] Fpocket uses Voronoi tessellation and α-spheres to identify distinct pockets within the protein structure. In this context, an α-sphere is defined as a sphere that touches four atoms on its boundary and contains no internal atoms. For consistency across all systems, we used the default Fpocket parameters, although for smaller pockets we generated grid maps with lower isovalues.

From these generated grid maps, we selected and saved a PDB file containing dummy atoms positioned at the grid points defining the regions of interest. Specifically, for each

system we selected a single pocket located within the cryptic site of the protein. These PDB files were later used as input to track the volume of the different pockets. A link to a PyMOL[11] session of each selected cryptic pocket is available in the Data Availability section. The volume values of the selected cryptic pockets are given in a table at the end of this section.

We analysed the behaviour of the selected pockets along the different biased and unbiased MD simulations using MDpocket, an open-source tool designed to detect binding pockets during MD simulations and part of the Fpocket 4.0 package.[10] MDpocket was applied to down-sampled and reference-aligned trajectories, with frames spaced at intervals of 100 ps. A suitable reference structure was carefully selected for each simulated system.

The output generated by MDpocket allowed us to measure the volume of the selected pockets along both biased and unbiased MD simulations. Visualisation of the resulting volume profiles was performed using different Python packages.

Volumes of the presented cryptic pockets.

**Protein**	**PDB ID (holo structure)**	**Cryptic Pocket Volume**†
TEM-1	1PZO	915 $Å^3$
Bcl-X_L	4C52	400 $Å^3$
IL-2	1PY2	415 $Å^3$
MDM2	5LAV	290 $Å^3$
HPV-11 E2	1R6N	155 $Å^3$

† = computed in the protonated and energy-minimised structure

1.4 Energy minimisation and equilibration protocol

Energy minimisation was performed over 50,000 steps using the steepest descent algorithm with a tolerance set at 100 kJ mol^{-1} nm^{-1}. The systems were equilibrated in three steps, with harmonic restraints applied to all heavy atoms only during the first two equilibration steps (harmonic constant: 1000 kJ mol^{-1} nm^{-1}). First, a 1 ns heating phase was performed in the NVT ensemble, using the V-rescale thermostat (τ = 0.1 ps).[12] Two different temperature coupling groups were used, one for the protein and the other including water molecules and ions, with a reference temperature of 300 K. A second 5 ns equilibration phase was then performed in the NPT ensemble using the V-rescale (τ = 0.5 ps) and Berendsen (P = 1 atm, τ = 0.5 ps) as thermostat and the barostat, respectively.[12,13] The same temperature coupling groups were maintained during the NPT equilibration step. An additional 5 ns equilibration step was performed in the NPT ensemble employing V-rescale (T = 300K, τ = 0.5 ps) and C-rescale (P = 1 atm, τ = 1 ps) as thermostat and barostat, respectively.

2 Effect of different initial data on the t-SNE embeddings

We tested the sensitivity of the t-SNE embeddings to the initial data points used to generate them. We performed different control simulations of the holo state of Bcl-X_L. We accumulated 750 ns of sampling for Bcl-X_L (initial structure PDB ID: 4C52) keeping the ligand in the pocket. Additionally, we accumulated 1.5 μs (3 replicas of 500 ns) of sampling of the holo-like structure of Bcl-X_L, obtained by removing the ligand from the pocket.

These control simulations allowed us to explore different pocket configurations: the holo control sampled only open configurations of the cryptic cavity, while the holo-like control explored not only the initial open conformations, but also semi-open and closed conformations. This is because, in the absence of the ligand, the cryptic site is free to partially (or completely) close.

We therefore generated a holo-like and a holo t-SNE embedding. For the holo-like embedding, we incorporated structures from our SWISH-X simulation of Bcl-X_L together with structures from the holo-like control simulations. For the holo embedding, we included structures from the SWISH-X simulation and structures from the holo control simulation. Clustering of these embeddings using HDBSCAN yielded a similar number of clusters (Figure S2: a-b).

We then calculated the Jaccard index to compare the similarity of the clusters obtained from the holo-like and the holo embedding (Figure S2c). The Jaccard index, denoted as $J(A, B)$, measures the similarity between two sets A and B. It is defined as the size of the intersection of the sets divided by the size of their union:

$$J(A, B) = \frac{|A \cap B|}{|A \cup B|}$$

where:

- $|A \cap B|$ represents the cardinality (number of elements) of the intersection of sets A and B.
- $|A \cup B|$ represents the cardinality of the union of sets A and B.

The Jaccard index ranges between 0 and 1, where:

- $J(A, B) = 0$ indicates that sets A and B have no elements in common.
- $J(A, B) = 1$ indicates that sets A and B have the same elements.

In most cases, any given cluster A from the holo-like embedding has a corresponding cluster B in the holo embedding such that $J(A, B) \sim 1$ (Figure S2c). For the cases where we have J values << 1, it is still possible to identify which clusters these structures end up in. For example, clusters 4 and 12 in Figure S2a are grouped into cluster 7 in Figure S2b, with $J(4, 7) = 0.26$ and $J(12, 7) = 0.71$ respectively (Figure S2c). Similarly, cluster 18 from Fig.S2a is assigned to the much larger cluster 19 in Figure S2b, hence $J(18, 19) \sim 0$. We

performed the same analysis on different embeddings and similar considerations apply (data not shown).

Taken together, these observations indicate that the majority of structures within a cluster in the holo-like embedding are consistently grouped into a single cluster in the holo embedding, and vice versa. This implies that similar structures sampled during the SWISH-X simulation tend to be consistently assigned to the same cluster, regardless of the original holo or holo-like control data used to create the embedding.

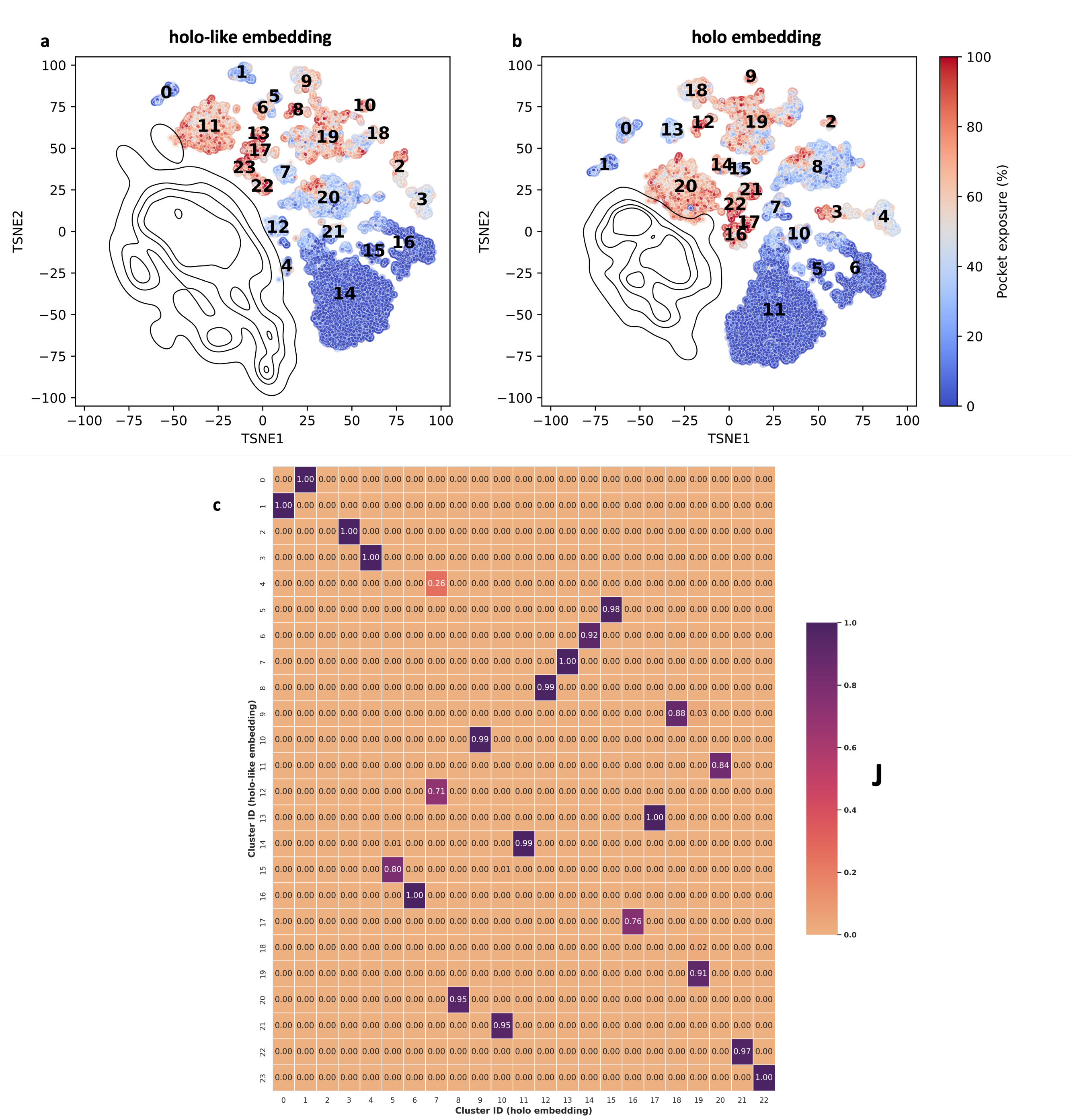

Figure S2: **t-SNE embeddings of Bcl-X$_L$.** t-SNE embeddings were generated from pocket configurations sampled during SWISH-X and holo-like (**a**) or holo simulations (**b**). Each t-SNE space displays coloured points corresponding to pocket configurations sampled during the SWISH-X simulation, with colours indicating the percentage of pocket exposure. Different numbers identify distinct clusters within each t-SNE cluster map. Isocontour lines delineate the regions explored by holo-like and holo simulations in *a* and *b* respectively. **c**) Jaccard index (J) for each holo-like and holo cluster pair from the t-SNE embeddings in *a* and *b*.

Table S1: Sampling time for each system presented.

Protein	PDB ID	Unbiased MD*	Mixed-Solvent*	SWISH-X**	SWISH**	Total
TEM-1	1JWP†	1 x 1 μs	3 x 500 ns	6 x 250 ns	6 x 250 ns	5.5 μs
	1PZO‡	3 x 500 ns				1.5 μs
$Bcl\text{-}X_L$	1R2D†	3 x 500 ns	3 x 500 ns	6 x 250 ns	6 x 250 ns	6 μs
	4C52‡	3 x 500 ns				1.5 μs
IL-2	1M47†	3 x 500 ns	3 x 500 ns	6 x 250 ns	6 x 250 ns	6 μs
	1PY2‡	3 x 500 ns				1.5 μs
MDM2	1Z1M†	3 x 500 ns	3 x 500 ns	5 x 250 ns	5 x 250 ns	5.5 μs
	5LAV‡	3 x 500 ns				1.5 μs
HPV-11 E2	1R6K†	3 x 500 ns	3 x 500 ns	6 x 250 ns	6 x 250 ns	6 μs
	1R6N‡	3 x 500 ns				1.5 μs

† = apo structure
‡ = holo-like structure
* = independent replicas
** = replica-exchange scheme

Table S2: SWISH-X and SWISH simulation parameters.

**Protein**	**PDB ID**	**Simulation type**	**Replicas**	**Exchange attempt rate***	**λ window**	**Opes Explore pace***	**Temperature range**
TEM-1	1JWP†	SWISH-X	6 x 250 ns	5000	1-1.35	5000	300-350 K
		SWISH	6 x 250 ns	5000	1-1.35	-	-
Bcl-X_L	1R2D†	SWISH-X	6 x 250 ns	2500	1-1.35	500	300-350 K
		SWISH	6 x 250 ns	5000	1-1.35	-	-
IL-2	1M47†	SWISH-X	6 x 250 ns	5000	1-1.35	5000	300-350 K
		SWISH	6 x 250 ns	5000	1-1.35	-	-
MDM2	1Z1M†	SWISH-X	5 x 250 ns	5000	1-1.28	5000	300-350 K
		SWISH	5 x 250 ns	5000	1-1.28	-	-
HPV-11 E2	1R6K†	SWISH-X	6 x 250 ns	5000	1-1.35	5000	300-330 K
		SWISH	6 x 250 ns	5000	1-1.35	-	-

† = apo structure

* = given as number of simulation steps ($\Delta t = 2\,\mathrm{fs}$)

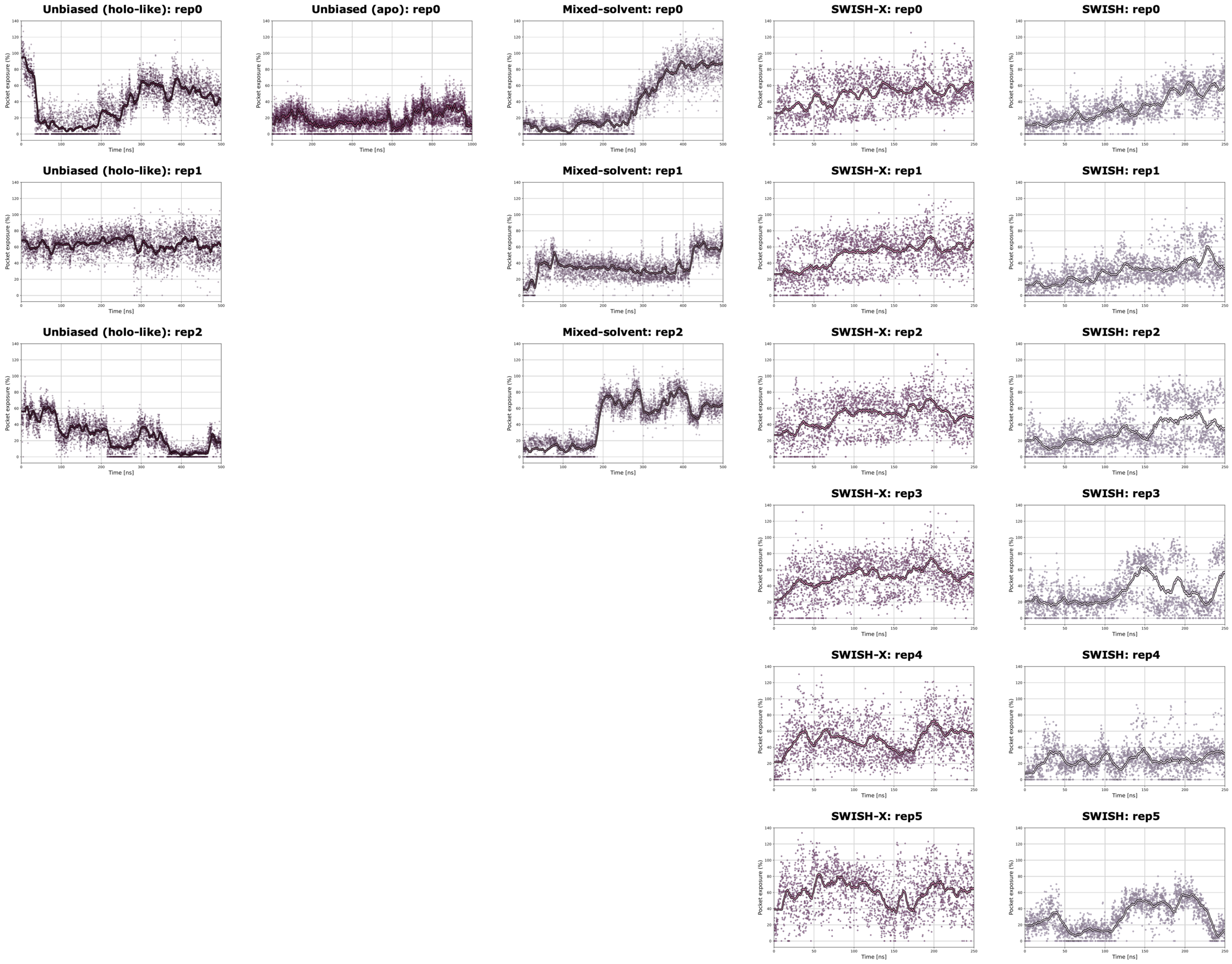

Figure S3: **Exposure of TEM-1's cryptic pocket in different simulations.** Comparison of the exposure profiles of TEM-1's cryptic site in unbiased MD (holo-like or apo), mixed-solvent, SWISH-X and SWISH simulations. Solid lines represent the running average of the pocket exposure over a 5 ns time window. Scatter points show the pocket exposure value for each structure in the simulation. Pocket exposure is expressed as a percentage of the volume of the cryptic site (Å^3) with respect to the volume of the corresponding pocket in the holo-crystal (PDB ID: 1PZO).

3 Effect of different tempering schemes on the opening of the cryptic pocket of TEM-1 β-lactamase

To further investigate the effect of temperature variations on the opening of TEM-1's cryptic pocket, we tested two different SWISH-X tempering schemes. The first, which we call SWISH-6X (yes, we ran out of imagination), gradually increases the temperature range explored by each replica (Table S3). The second scheme we tested is identical to that used in SWISH-X, with each replica covering the same temperature range, but we broadened the temperature range from 300-350 K to 280-350 K. In both cases, the resulting pocket sampling from both SWISH-6X and SWISH-X (280-350 K) is not as efficient as in SWISH-X at higher temperatures, with a median site opening of 33%, 38% and 53% respectively (Figure S4a). In addition, comparison of the volume profiles of each replica clearly shows how the higher temperature range greatly improves the opening of the pocket (Figure S4 b-c).

Table S3: Different tempering schemes tested for TEM-1 β-lactamase.

**Simulation type**	**Replicas**	**Temperature Rep 1**	**Temperature Rep 2**	**Temperature Rep 3**	**Temperature Rep 4**	**Temperature Rep 5**	**Temperature Rep 6**
SWISH-X	6 x 250 ns	300-350 K	300-350 K	300-350 K	300-350 K	300-350 K	300-350 K
SWISH-X	6 x 250 ns	280-350 K	280-350 K	280-350 K	280-350 K	280-350 K	280-350 K
SWISH-6X	6 x 250 ns	300K	300-310K	300-320K	300-330 K	300-340K	300-350 K

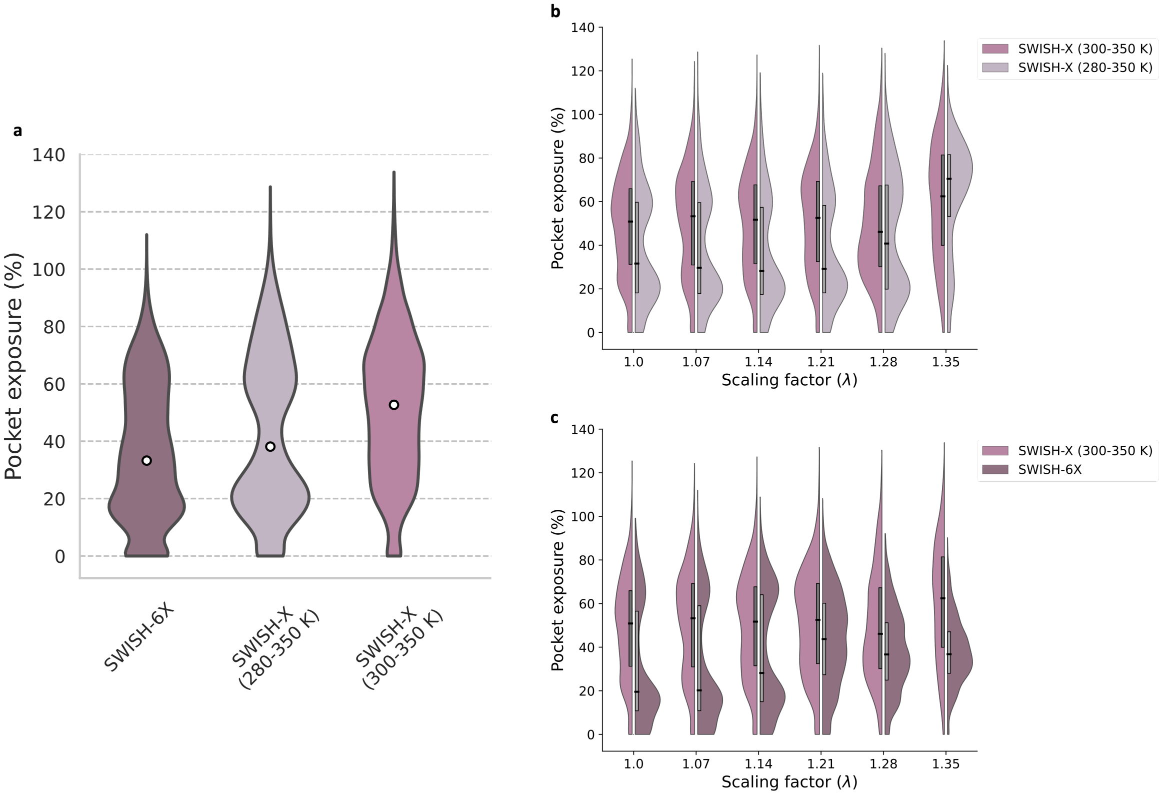

Figure S4: **Opening of the cryptic pocket of TEM-1 under different tempering schemes.** **a**) Violin plots of the cryptic site exposure of the selected cryptic pocket along different simulations. The simulations are presented as follows (from left to right): SWISH-6X, SWISH-X (280-350 K) and SWISH-X (300-350 K). Pocket exposure values from individual replicas of a given simulation are combined into a single violin plot. **b**) Comparison of pocket exposure profiles along different replicas of TEM-1's SWISH-X simulations sampling different temperature ranges. **c**) Comparison of the pocket exposure profiles along different replicas of the SWISH-X and SWISH-6X simulations of TEM-1. Pocket exposure is expressed as a percentage of the volume of the cryptic site ($Å^3$) with respect to the volume of the corresponding pocket in the holo-crystal (PDB ID: 1PZO).

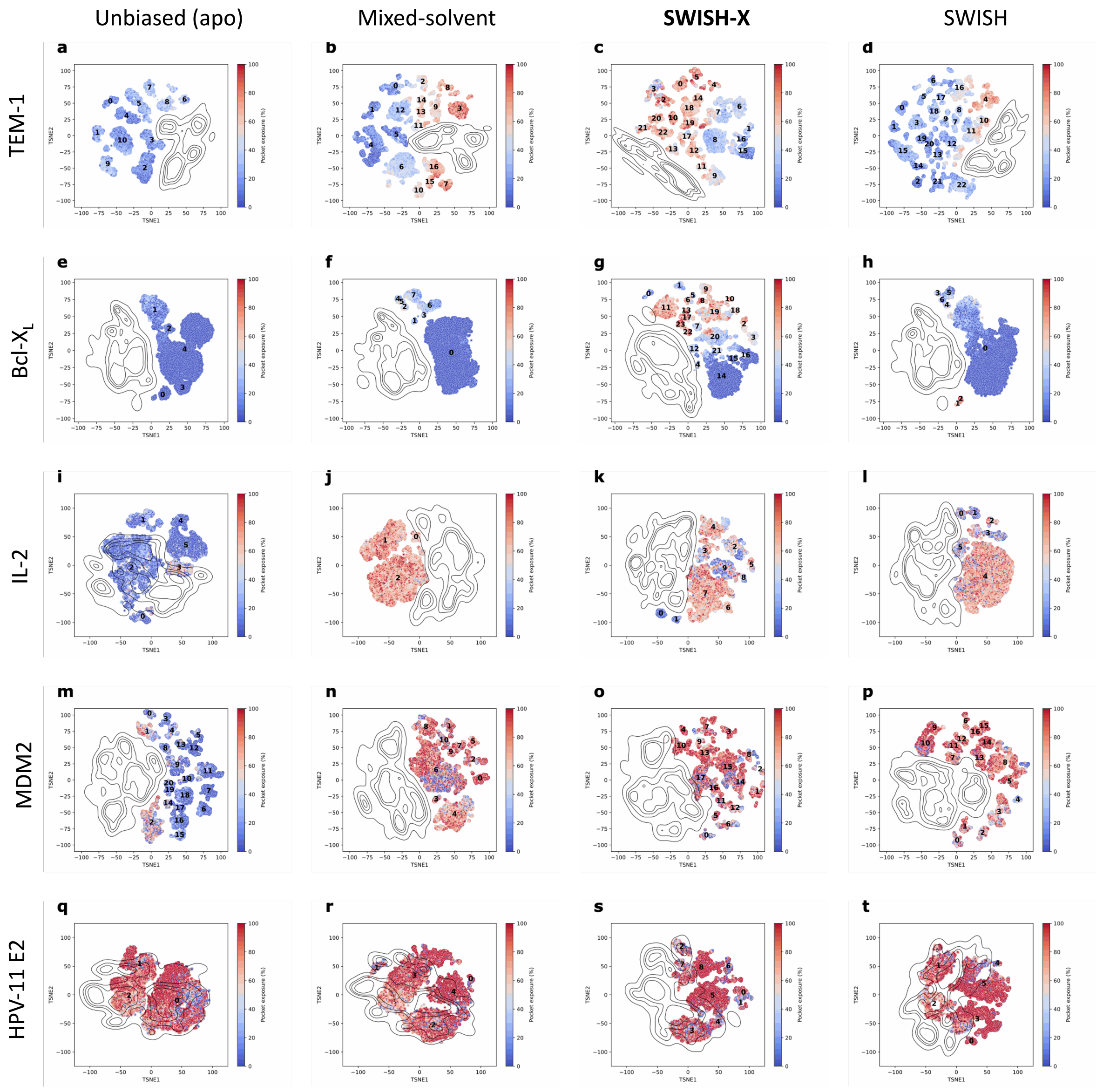

Figure S5: **t-SNE volume maps of the different cryptic pockets.** Each point in a given t-SNE space corresponds to a pocket configuration sampled during either an apo unbiased, SWISH-X, mixed-solvent or SWISH simulation, with colours indicating the percentage of pocket exposure. Different numbers identify distinct clusters within each t-SNE cluster map. Isocontour lines highlight the region of the t-SNE space explored by holo-like simulations.

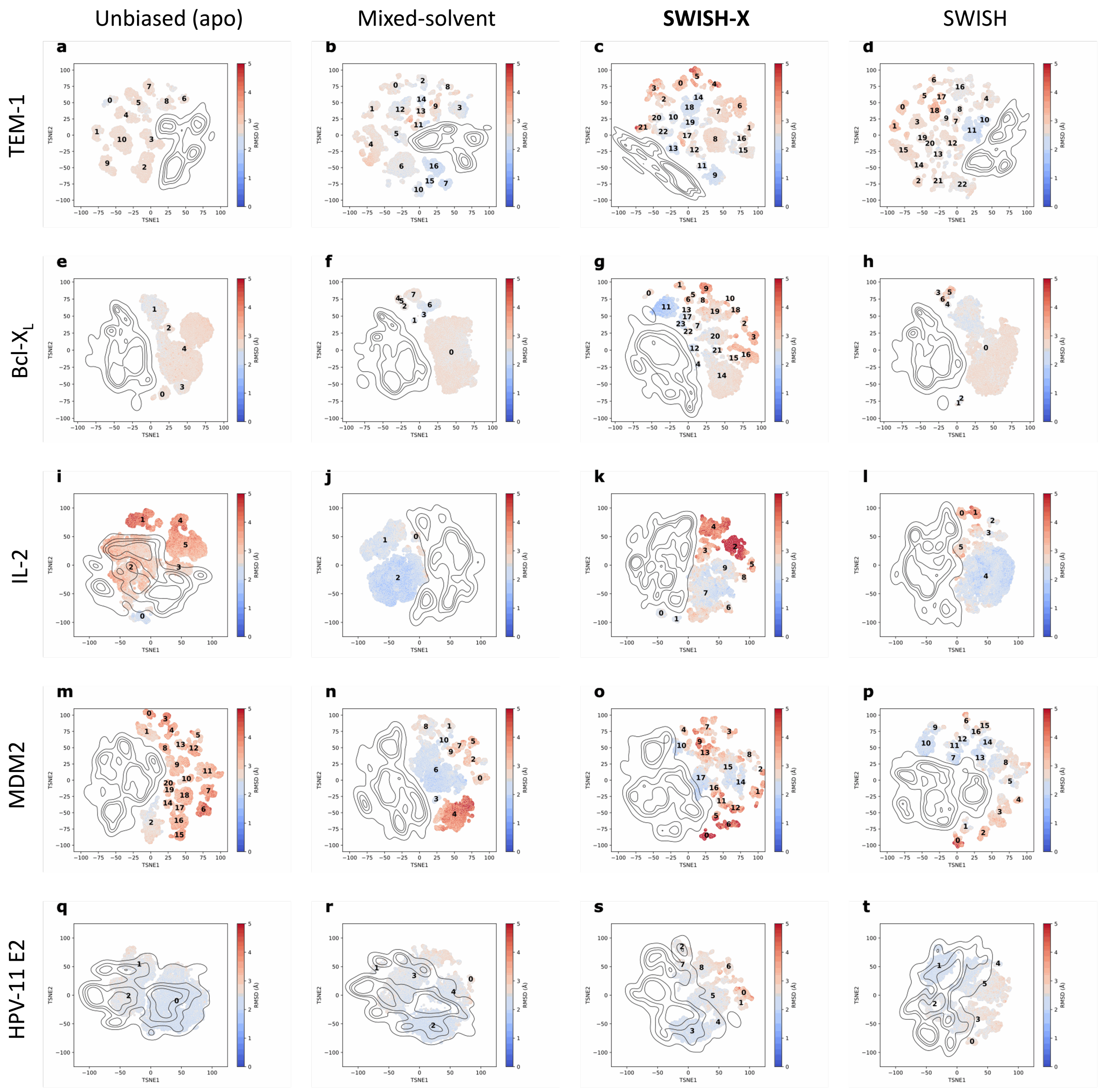

Figure S6: **t-SNE RMSD maps of the different cryptic pockets.** Each point in a given t-SNE space corresponds to a pocket configuration sampled during either an apo unbiased, SWISH-X, mixed-solvent or SWISH simulation, with colours indicating RMSD values ($\mathring{A}$). The RMSD was calculated as the heavy atom RMSD with respect to the residues located within 7 $\mathring{A}$ of the centre of the cryptic pocket in the corresponding holo crystal. Different numbers identify distinct clusters within each t-SNE cluster map. Isocontour lines highlight the region of the t-SNE space explored by holo-like simulations.

4 Opening times of the different cryptic pockets

We calculated the opening times of the different cryptic cavities based on the different sampling schemes we tested (Table S4). We defined two opening thresholds, corresponding to 60% and 80% exposure of the cryptic pocket with respect to the corresponding holo-crystal. For any given simulation, we selected only structures that met these opening thresholds. We then filtered out high RMSD pocket configurations (RMSD > 2 $\AA$) with respect to their corresponding holo-crystal configuration. The RMSD of a given pocket structure was calculated with respect to all heavy atoms of the residues within 7 $\AA$ from the centre of the pocket in the holo-crystal. Subsequently, we checked how long it took for each simulation strategy to sample at least one structure that met these criteria. This provided us with a quantitative indication of how long it takes each simulation to sample an open-crystal-like configuration of the cryptic site.

Table S4: Cryptic pockets opening times under different sampling schemes.

	**60% exposure**			**80% exposure**		
**Protein**	**Mixed-Solvent**	**SWISH-X**	**SWISH**	**Mixed-Solvent**	**SWISH-X**	**SWISH**
TEM-1	181.7 ns	24.3 ns	95.8 ns	195.8 ns	24.3 ns	176 ns
Bcl-X_L	214.0 ns	56.2 ns	227 ns	-	56.6 ns	227 ns
IL-2	<5 ns	<5 ns	<5 ns	<5 ns	<5 ns	<5 ns
MDM2	22.1 ns	1.7 ns	8 ns	22.1 ns	1.7 ns	8 ns
HPV-11 E2	5.2 ns	3.5 ns	12.6 ns	5.2 ns	4.0 ns	15.6 ns

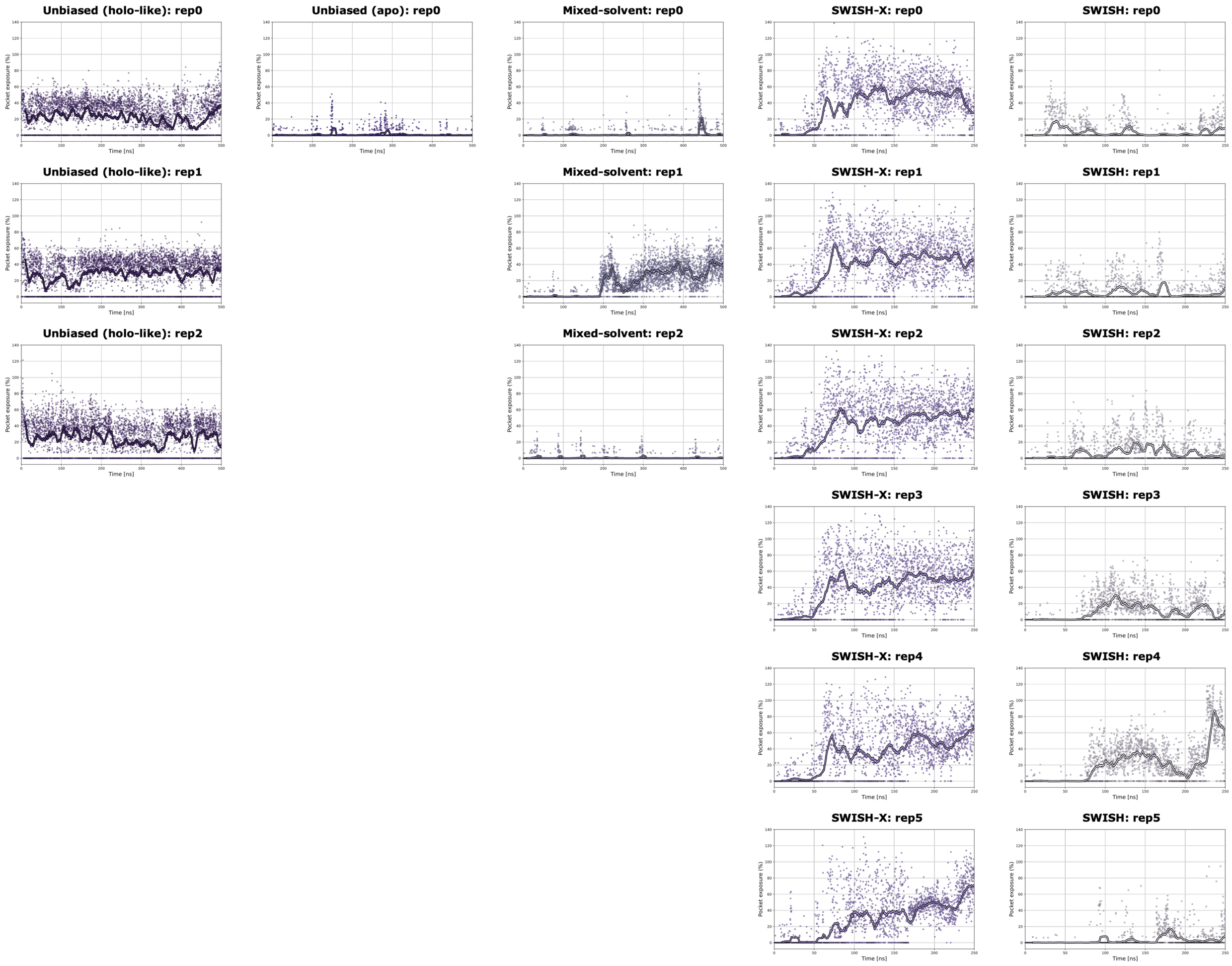

Figure S7: **Exposure of Bcl-X$_L$'s cryptic pocket in different simulations.** Comparison of the exposure profiles of Bcl-X$_L$'s cryptic site in unbiased MD (holo-like or apo), mixed-solvent, SWISH-X and SWISH simulations. Solid lines represent the running average of the pocket exposure over a 5 ns time window. Scatter points show the pocket exposure value for each structure in the simulation. Pocket exposure is expressed as a percentage of the volume of the cryptic site (Å^3) with respect to the volume of the corresponding pocket in the holo-crystal (PDB ID: 4C52).

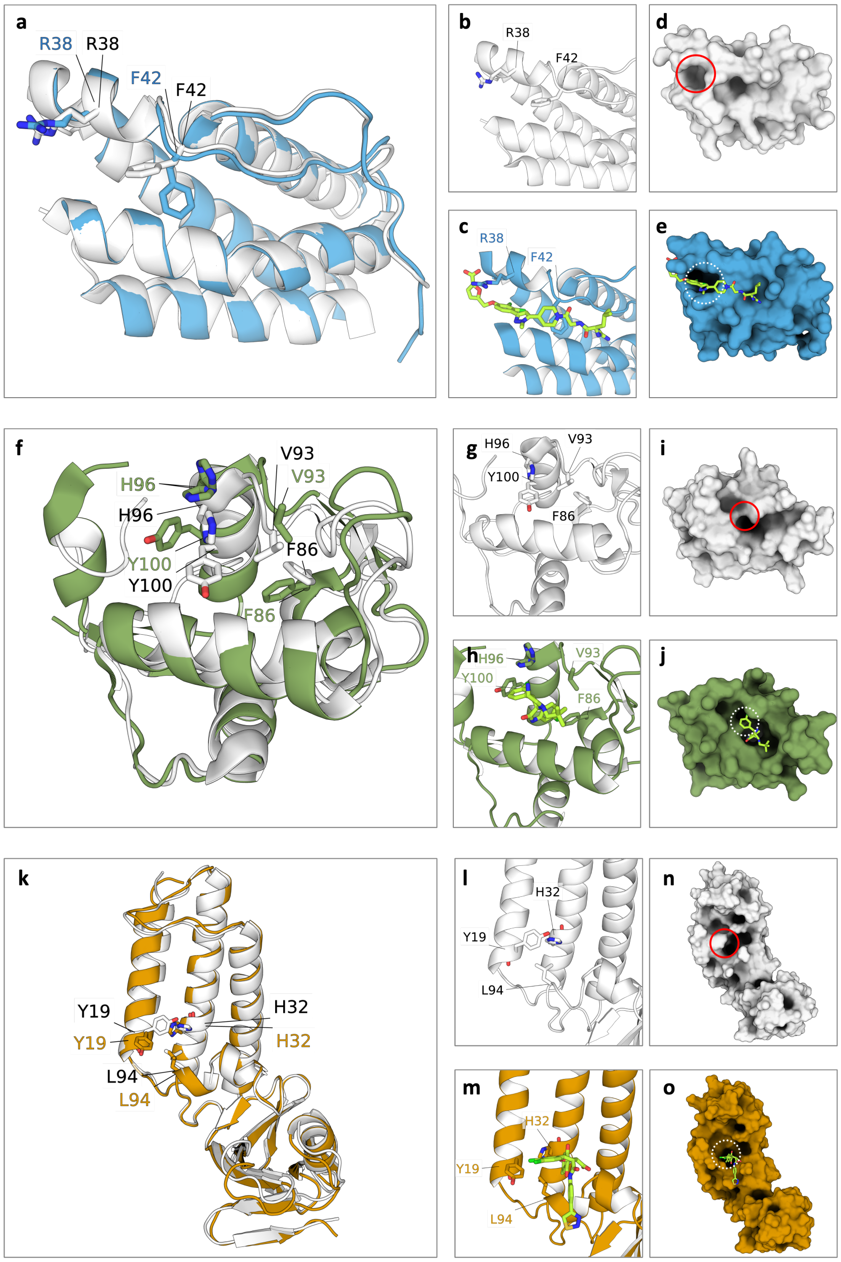

Figure S8: **Cryptic pockets in three different PPI systems. a)** Structural alignment of IL-2's apo (white, PDB ID: 1M47) and holo (blue, PDB ID: 1PY2) structures. **b-c)** Close-up views of the cryptic cavity in apo and holo structures, respectively. **d-e)** Surface representation of apo (white) and holo IL-2 (blue). **f)** Structural alignment of MDM2's apo (white, PDB ID: 1Z1M) and holo (green, PDB ID: 5LAV) structures. **g-h)** Close-up views of the cryptic cavity in apo and holo structures, respectively. **i-j)** Surface representation of apo (white) and holo MDM2 (green). **k)** Structural alignment of HPV-11 E2's apo (white, PDB ID: 1R6K) and holo (orange, PDB ID: 1R6N) structures. **l-m)** Close-up views of the cryptic cavity in apo and holo structures. **n-o)** Surface representation of apo (white) and holo HPV-11 E2 (orange). Relevant residues are depicted as sticks and labelled, with the inhibitor omitted for clarity (panels *a*, *f* and *k*). Inhibitors are shown as green sticks (panels *c*, *h* and *m*). The cryptic pockets are detectable in the holo structures (dotted white circles in panels *e*, *j* and *o*) but not in the corresponding apo structures (solid red circles in panels *d*, *i* and *n*).

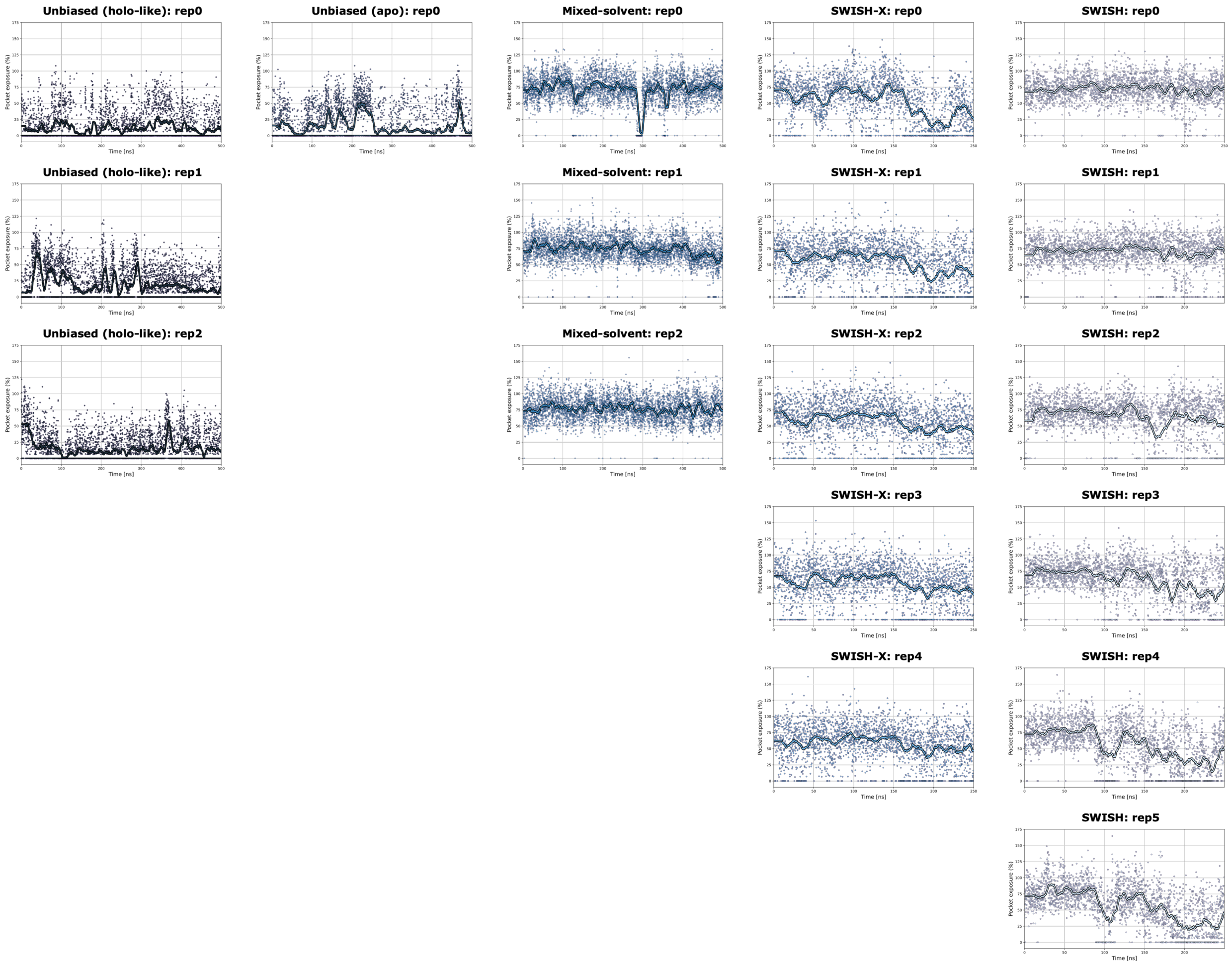

Figure S9: **Exposure of IL-2's cryptic pocket in different simulations.** Comparison of the exposure profiles of IL-2's cryptic site in unbiased MD (holo-like or apo), mixed-solvent, SWISH-X and SWISH simulations. Solid lines represent the running average of the pocket exposure over a 5 ns time window. Scatter points show the pocket exposure value for each structure in the simulation. Pocket exposure is expressed as a percentage of the volume of the cryptic site (Å^3) with respect to the volume of the corresponding pocket in the holo-crystal (PDB ID: 1PY2).

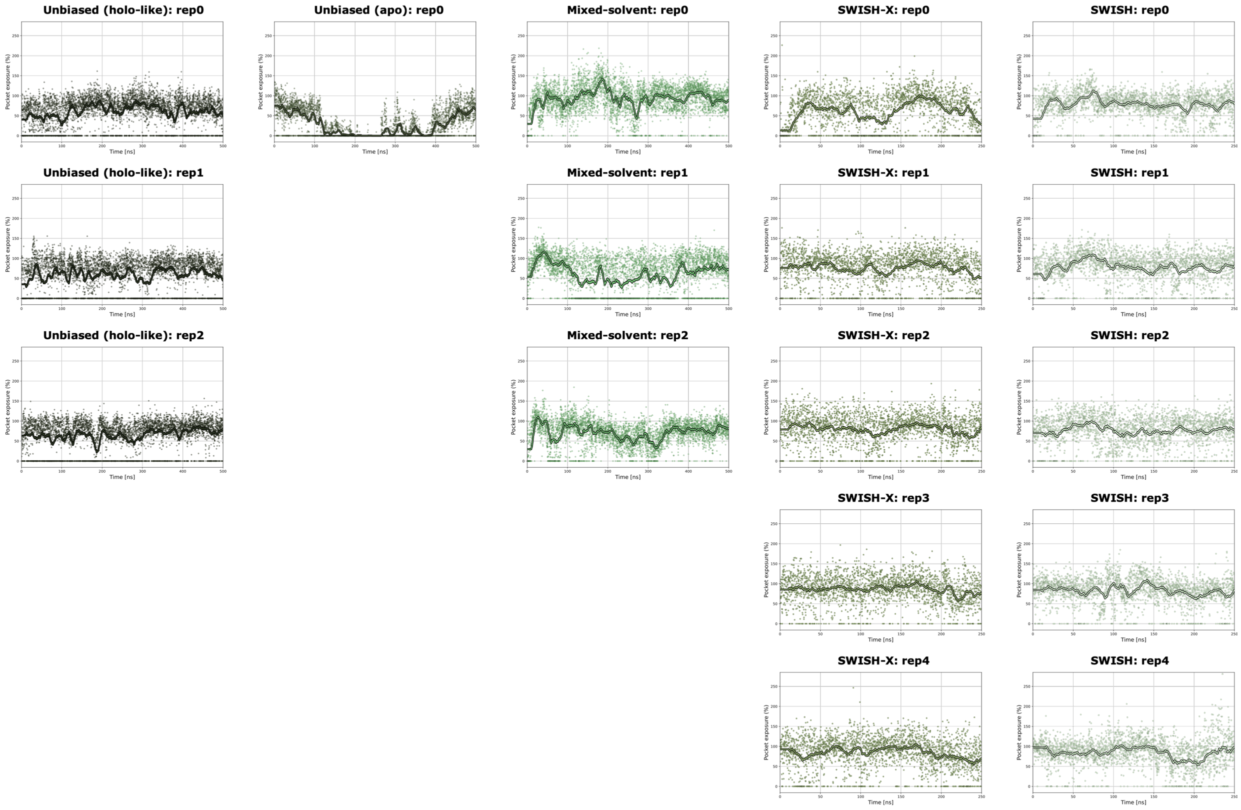

Figure S10: **Exposure of MDM2's cryptic pocket in different simulations.** Comparison of the exposure profiles of MDM2's cryptic site in unbiased MD (holo-like or apo), mixed-solvent, SWISH-X and SWISH simulations. Solid lines represent the running average of the pocket exposure over a 5 ns time window. Scatter points show the pocket exposure value for each structure in the simulation. Pocket exposure is expressed as a percentage of the volume of the cryptic site (Å^3) with respect to the volume of the corresponding pocket in the holo-crystal (PDB ID: 5LAV).

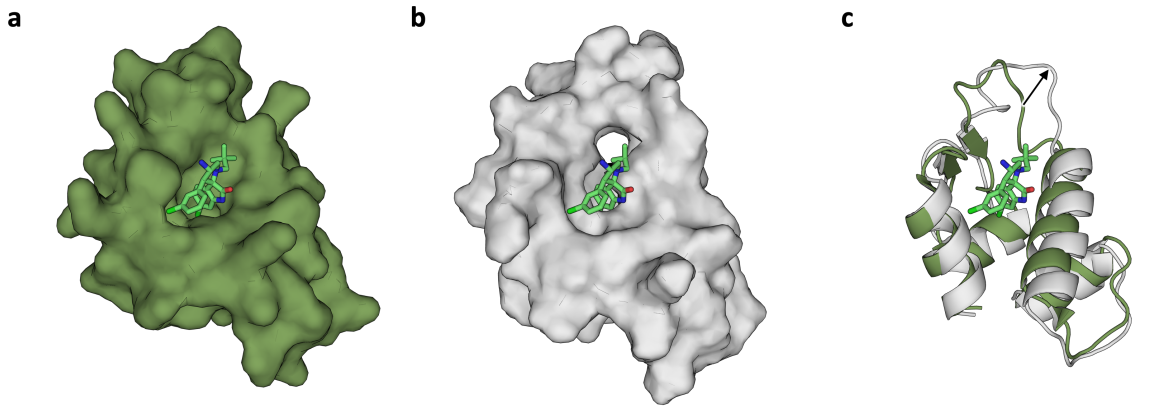

Figure S11: **Example of novel open conformation in MDM2's SWISH-X simulation.** **a)** Inhibitor-bound X-ray structure (PDB ID: 5LAV), the protein is represented as surface, the ligand is shown as green sticks. **b)** Representative structure from the SWISH-X simulation (ligand from PDB ID: 5LAV was included for reference). **c)** Secondary structure alignment between the X-ray inhibitor-bound MDM2 (green) and a representative conformation from the SWISH-X simulation (grey). The arrow illustrates the loop movement required to expose the deeper cavity.

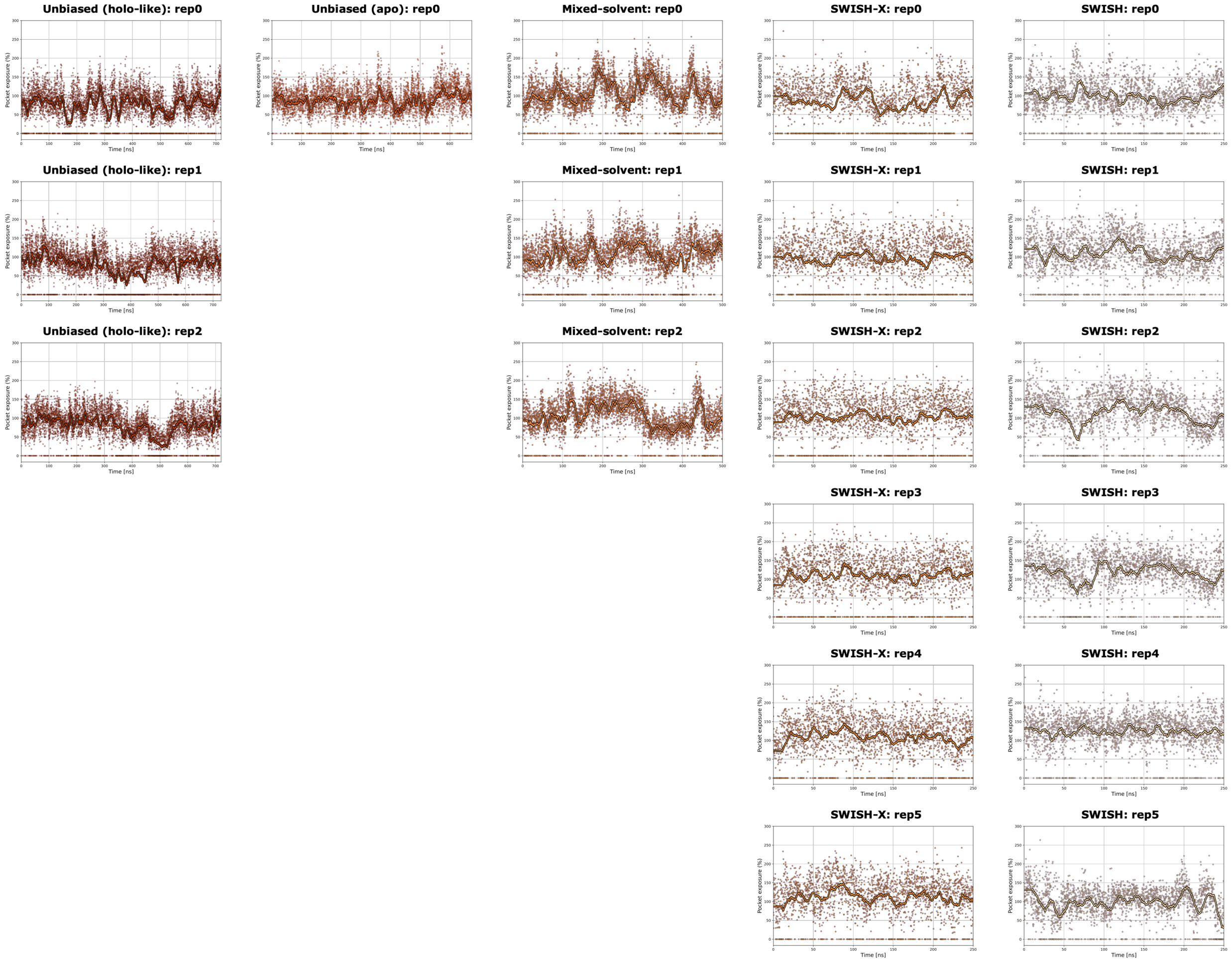

Figure S12: **Exposure of HPV-11 E2's cryptic pocket in different simulations.** Comparison of the exposure profiles of HPV-11 E2's cryptic site in unbiased MD (holo-like or apo), mixed-solvent, SWISH-X and SWISH simulations. Solid lines represent the running average of the pocket exposure over a 5 ns time window. Scatter points show the pocket exposure value for each structure in the simulation. Pocket exposure is expressed as a percentage of the volume of the cryptic site (Å^3) with respect to the volume of the corresponding pocket in the holo-crystal (PDB ID: 1R6N).

References

(1) Li, M.; Wang, D.; He, J.; Chen, L.; Li, H. Bcl-XL: A multifunctional anti-apoptotic protein. *Pharmacological Research* **2020**, *151*, 104547.

(2) Manion, M. K.; O'Neill, J. W.; Giedt, C. D.; Kim, K. M.; Zhang, K. Y.; Hockenbery, D. M. Bcl-XL mutations suppress cellular sensitivity to antimycin A. *Journal of Biological Chemistry* **2004**, *279*, 2159–2165.

(3) Lee, E. F.; Grabow, S.; Chappaz, S.; Dewson, G.; Hockings, C.; Kluck, R. M.; Debrincat, M. A.; Gray, D. H.; Witkowski, M. T.; Evangelista, M., et al. Physiological restraint of Bak by Bcl-xL is essential for cell survival. *Genes & development* **2016**, *30*, 1240–1250.

(4) Boyman, O.; Sprent, J. The role of interleukin-2 during homeostasis and activation of the immune system. *Nature Reviews Immunology* **2012**, *12*, 180–190.

(5) Tilley, J. W.; Chen, L.; Fry, D. C.; Emerson, S. D.; Powers, G. D.; Biondi, D.; Varnell, T.; Trilles, R.; Guthrie, R.; Mennona, F., et al. Identification of a small molecule inhibitor of the IL-2/IL-2Rα receptor interaction which binds to IL-2. *Journal of the American Chemical Society* **1997**, *119*, 7589–7590.

(6) Thanos, C. D.; DeLano, W. L.; Wells, J. A. Hot-spot mimicry of a cytokine receptor by a small molecule. *Proceedings of the National Academy of Sciences* **2006**, *103*, 15422–15427.

(7) Wang, S.; Chen, F.-E. Small-molecule MDM2 inhibitors in clinical trials for cancer therapy. *European Journal of Medicinal Chemistry* **2022**, 114334.

(8) Gollner, A.; Rudolph, D.; Arnhof, H.; Bauer, M.; Blake, S. M.; Boehmelt, G.; Cockroft, X.-L.; Dahmann, G.; Ettmayer, P.; Gerstberger, T., et al. Discovery of novel spiro

[3 H-indole-3, 2-pyrrolidin]-2 (1 H)-one compounds as chemically stable and orally active inhibitors of the MDM2-p53 interaction. *Journal of medicinal chemistry* **2016**, *59*, 10147–10162.

(9) Wang, Y.; Coulombe, R.; Cameron, D. R.; Thauvette, L.; Massariol, M.-J.; Amon, L. M.; Fink, D.; Titolo, S.; Welchner, E.; Yoakim, C., et al. Crystal structure of the E2 transactivation domain of human papillomavirus type 11 bound to a protein interaction inhibitor. *Journal of Biological Chemistry* **2004**, *279*, 6976–6985.

(10) Le Guilloux, V.; Schmidtke, P.; Tuffery, P. Fpocket: an open source platform for ligand pocket detection. *BMC bioinformatics* **2009**, *10*, 1–11.

(11) Schrodinger, L. The PyMOL molecular graphics system. *Version* **2015**, *1*, 8.

(12) Bussi, G.; Donadio, D.; Parrinello, M. Canonical sampling through velocity rescaling. *The Journal of chemical physics* **2007**, *126*, 014101.

(13) Berendsen, H. J.; Postma, J. v.; Van Gunsteren, W. F.; DiNola, A.; Haak, J. R. Molecular dynamics with coupling to an external bath. *The Journal of chemical physics* **1984**, *81*, 3684–3690.
